# Comparative single‐cell transcriptomic analysis across tissues of aging primates reveals specific autologous activation of ZNF281 to mitigate oxidative stress in cornea

**DOI:** 10.1111/acel.14319

**Published:** 2024-09-10

**Authors:** Yuhua Xiao, Xu Chen, Zheyao Chen, Wangxuan Dai, Xing Hu, Shuyao Zhang, Jiawei Zhong, Jia Chen, Xu Liu, Lingyi Liang, Youjin Hu

**Affiliations:** ^1^ State Key Laboratory of Ophthalmology, Zhongshan Ophthalmic Center Sun Yat‐Sen University, Guangdong Provincial Key Laboratory of Ophthalmology Visual Science Guangzhou China

**Keywords:** aging, corneal epithelium, nonhuman primate, reactive oxygen species, single‐cell transcriptomic analysis

## Abstract

Reactive oxygen species (ROS) and oxidative stress accelerate cellular aging, but their impact on different tissues varies. The cornea, known for its robust antioxidant defense systems, is relatively resistant to age‐related diseases like cancer. However, the precise mechanisms by which the cornea maintains ROS homeostasis during aging remain unclear. Through comparative single‐cell transcriptomic analysis of the cornea and other tissues in young and old nonhuman primates, we identified that a ZNF281 coding transcriptomic program is specifically activated in cornea during aging. Further investigation revealed that ZNF281 forms a positive feedback loop with FOXO3 to sense elevated levels of ROS and mitigate their effects potentially by regulating the mitochondrial respiratory chain and superoxide dismutase (SOD) expression. Importantly, we observed that overexpression of ZNF281 in MSCs prevented cellular senescence. In summary, these findings open up possibilities for understanding tissue‐specific aging and developing new therapies targeting ROS damage.

## Graphical abstract

As cells age, there is an increase in the production of reactive oxygen species (ROS), leading to oxidative stress. ZNF281 and FOXO3 serve as sensors for ROS and mutually enhance their expression levels. Consequently, the elevated expression of ZNF281 and FOXO3 leads to an increase in the expression of antioxidant genes, such as SOD1 and SOD2, while downregulating genes related to oxidative phosphorylation (OXPHOS) in mitochondria and those that govern mitochondrial biogenesis. This coordinated response effectively reduces cellular ROS levels, thereby maintaining cellular homeostasis during the aging process.

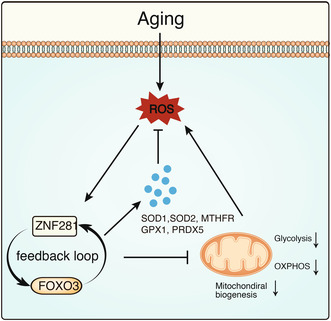

**Abbreviations**
BCs: basal cells
BSA: bovine serum albumin
CCA: canonical Correlation Analysis
CUT&Tag: cleavage under targets and tagmentation
DCFH‐DA: dichlorodihydrofluorescein diacetate
DEGs: differentially expressed genes
ECAR: extracellular acidification rate
GEMs: gel beads in emulsion
GEO: gene Expression Omnibus
GO: gene ontology
GSA: genome Sequence Archive
hCECs: human corneal epithelial cells
HFF: human foreskin fibroblast
HSCs: hematopoietic stem cells
IACUC: institutional animal care and use committee
MOI: multiplicity of infection
MSCs: mesenchymal stem cells
NSCs: neural stem cells
OCR: oxygen consumption rate
OXPHOS: oxidative phosphorylation
PCA: principal component analysis
ROS: reactive oxygen species
RPE: retinal pigment epithelium
RT‐qPCR: real‐time quantitative PCR
SA‐β‐gal: senescence‐associated β‐galactosidase
scRNA‐seq: single‐cell level RNA sequencing
SOD: superoxide dismutase
TF: transcription factors

## 1 INTRODUCTION

The accumulation of reactive oxygen species (ROS) and oxidative stress contributes to the aging phenotype and affects longevity (Finkel & Holbrook, 2000), while the antioxidant defenses vary across different tissues (Corona et al., 2007; Seehuus et al., 2006). For instance, human facial skin, due to frequent UV exposure, is highly prone to aging‐associated alterations (Flament et al., 2013) such as reduced epithelial thickness and wrinkling (Zou et al., 2021), and experiences a high incidence of skin cancer. Interestingly, the corneal epithelium, also exposed to high UV radiation, shows orders of magnitude lower susceptibility to aging‐related diseases such as cancer (Cardenas‐Cantu et al., 2016; Faragher et al., 1997). Identifying the mechanisms that maintain ROS homeostasis and corneal function with aging may provide valuable information for antiaging treatments and prevention of age‐related diseases.

During aging, oxidative stress usually results from either excessive ROS production, mitochondrial dysfunction, impaired antioxidant system, or a combination of these factors (Nita & Grzybowski, 2016). Numerous transcription factors (TFs) have been identified in previous studies to be involved in regulating ROS homeostasis. Cells that exhibit increased resistance to aging often undergo transcriptomic reprogramming, which includes the upregulation of genes implicated in ROS sensing and scavenging (Stoeger et al., 2022). An example of such a transcription factor is FOXO3, which plays a key role in regulating the transcription of genes associated with ROS sensing and scavenging. Sustained expression of FOXO3 has been demonstrated to extend the lifespan of aging model organisms by enhancing peroxisomal functions (Hayes & Dinkova‐Kostova, 2014; Sies & Jones, 2020). The decline of FOXO3 and other TF regulating ROS homeostasis in certain tissues, such as arteries, is a significant factor in the aging process. However, it remains unclear how the transcriptome is autologously reprogrammed in the cornea to protect it from ROS‐induced oxidative stress, given that the majority of these TF decrease with age.

Nonhuman primates are an ideal model for studying aging‐related diseases due to their genetic, anatomical, and physiological similarities to humans, including sensitivity to aging‐related diseases (Wang et al., 2020; Zhang et al., 2020). In this study, we constructed single‐cell atlases across four organs from young and old nonhuman primates and revealed tissue‐and cell‐specific transcriptomic alterations involved in aging. Through comparative single‐cell transcriptomic analysis of the cornea and other tissues in young and old nonhuman primates, we identified that a ZNF281 coding program is specifically activated in cornea during aging. Further investigation revealed that ZNF281 forms a positive feedback loop with FOXO3 to sense increased levels of ROS and mitigate their effects potentially by regulating the mitochondrial respiratory chain and superoxide dismutase (SOD) expression. Overexpression of ZNF281 in MSCs prevented cellular senescence. In summary, these findings open up possibilities for understanding tissue‐specific aging and developing new therapies targeting ROS damage.

## 2 RESULTS

### 2.1 Single‐cell transcriptomic analysis uncovers the characteristics of the aging corneal epithelium

**FIGURE 1:**
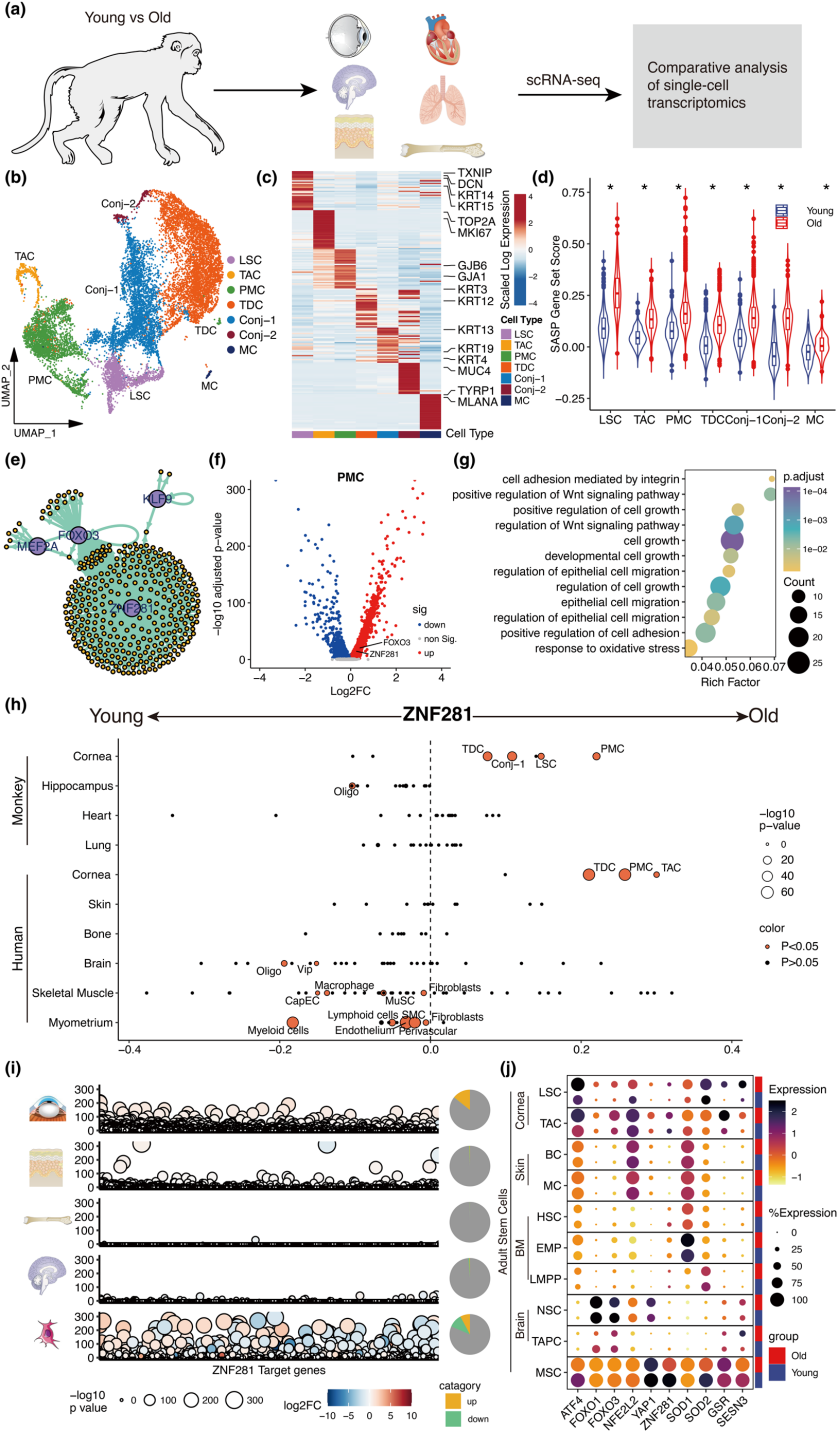
Single‐cell transcriptomic analysis uncovers the characteristics of the aging corneal epithelium. (a) The schematic representation showing the single‐cell RNA sequencing (scRNA‐seq) analysis pipeline, which includes a comparative study of aging changes in the monkey corneal epithelium and across different organs (including hippocampus, lung, heart from monkeys, and bone and skin from humans). This representation also compares the aging process in the corneal epithelium across various species. (b) UMAP plot showing the seven cell types comprising primate corneal epithelium. LSCs, limbal stem cells; TACs, transit amplifying cells; MKI67+, PMCs, postmitotic cell; TDCs, terminally differentiated cells; Conj‐1, conjunctival epithelial cells‐1; Conj‐2, conjunctival epithelial cells‐2; MCs, melanocytes. (c) Heatmap showing the expression signature of the top 30 marker genes for each identified cell type. (d) Violin plots of gene set scores related to the SASP in young and aged groups. (e) Network visualization of core regulatory TFs in the 7 cell types in corneal epithelium between the old and young groups. (f) Volcano plots indicating that aging upregulates ZNF281 and FOXO3 in PMC of the corneal epithelium. (g) Dot plot showing the enriched GO term of ZNF281 target genes. (h) Dot plots showing the variation in *ZNF281* expression during aging across different organ. Oligo, oligodendrocyte; Vip, VIP+ neuron; MuSC, muscle stem cell; CapEC, Capillary endothelial cell; SMC, smooth muscle cell. (i) Bubble plot showing the expression of ZNF281 target genes is uniquely upregulated in the stem cell in aging corneal epithelium. (j) Dot plot showing genes exhibiting oxidant capacity are specifically upregulated within stem cells in the aging corneal epithelium.

Single‐cell level RNA sequencing (scRNA‐seq) was performed on corneal epithelium samples from both a young (4‐year‐old) and an older (19‐year‐old) cynomolgus monkey to identify cell type‐specific gene expression profiles and examine how these profiles change with age (Figure 1a). Following rigorous cell quality assessment and filtering, a total of 14,058 high‐quality cells were retained for downstream analysis (Figure 1b). Canonical correlation analysis (CCA) combined with unsupervised clustering identified seven distinct clusters exhibiting unique cellular transcriptomic signatures: limbal stem cells (LSCs; KRT14+, KRT15+, MMP1+, GPHA2+), transit amplifying cells (TACs; MKI67+, TOP2A+), postmitotic cells (PMCs; GJB6+, GJA1+), terminally differentiated cells (TDCs; KRT3+, KRT12+), conjunctival epithelial cells‐1 (Conj‐1; MUC1+, MUC4+), conjunctival epithelial cells‐2 (Conj‐2; KRT13+, KRT4+, KRT19+), and melanocytes (MCs; TYRP1+, MLANA+) (Figure 1b,c).

To more comprehensively describe the changes in epithelial cell characteristics during aging, we compared cell proportion and gene expression profiles between the young and aging corneal epithelial cells. Corneal epithelium cells from the aging monkey showed reduced fractions of LSCs and Conj‐2, and larger fractions of TACs and PMCs (Figure S1a). Among cyclin‐dependent kinase inhibitors (CKIs) of the Cip/Kip and INK4 families (Figure S1b,c), the p21‐encoding CDKN1A gene, a marker of aging and cellular senescence (Huang et al., 2022), was found to be upregulated in most aging corneal epithelial cell subtypes.

Analysis of average gene set expression profiles using Seurat revealed that the senescence‐associated secretory phenotype (SASP) gene set was globally upregulated in the aged corneal epithelium (Figure 1d). This upregulation was also observed in other age‐related gene sets, including response to ROS and proteostasis, while downregulation was observed in DNA repair (Figure S1f–h). We further investigated transcriptional alterations associated with aging at the cellular level. In total, 231 genes were upregulated and 139 downregulated in aged LSCs versus younger LSCs, while 179 were upregulated and 95 downregulated in older TACs, 240 upregulated and 124 downregulated in older PMCs, and 199 upregulated and 138 downregulated in older TDCs (Figure S1d,e). According to GO enrichment analysis, upregulated differentially expressed genes (DEGs) are associated with “regulation of apoptotic signaling pathway” and “negative regulation of response to wounding”, indicating a diminished capacity for corneal self‐renewal and repair with aging (Figure S1i), while downregulated DEGs are associated with response to wounding, also reflecting a diminished capacity for corneal self‐renewal and repair (Figure S1j).

To elucidate the gene regulatory network involved in corneal aging, we performed a transcriptional regulatory network analysis. This analysis identified several TFs, including FOXO3 and ZNF281 (Figure 1e), which are predominantly upregulated in most aging corneal cell types (Figure 1f). These TFs were identified as core regulatory TFs in the transcriptional network constructed by SCENIC, suggesting their potential role in regulating the aging process of corneal cells. Subsequently, we performed a Gene Ontology (GO) enrichment analysis of ZNF281 target genes that were differentially expressed in the aging cornea. The results indicated that ZNF281 might regulate cell growth, cell migration, and response to oxidative stress in aging corneal cells (Figure 1g).

### 2.2 Aging corneal epithelium displays a targeted activation of a specialized antioxidant transcriptional program regulated by ZNF281 and FOXO3

To further understand the role of ZNF281 in organ aging, we performed a comparative single‐cell transcriptomic analysis between the cornea and other aging organs, including heart, lung (Ma et al., 2021), and hippocampus (Zhang et al., 2021) from monkeys, using publicly single‐cell RNA sequencing datasets (Figure S2). Intriguingly, ZNF281 exhibited a distinctive upregulation in the aging cornea, despite no significant difference in its baseline expression between the cornea and other organs in monkeys (Figure 1h; Figure S3a–d). FOXO3 displayed a similar expression change pattern across different organ aging processes, akin to ZNF281, with the exception of its upregulation in excitatory neurons (ExN) cells from the hippocampus (Figure S3e). Furthermore, we analyzed the expression changes of ZNF281 and FOXO3 in aging human cornea (Li et al., 2021; Maiti et al., 2022), as well skin (Zou et al., 2021), bone marrow (Oetjen et al., 2018), brain (Emani et al., 2024), skeletal muscle (Kedlian et al., 2024), myometrium (Punzon‐Jimenez et al., 2024). Consistent with our findings in monkeys, ZNF281 was demonstrated to be upregulated in the cornea while downregulated in other aging organs, such as skeletal muscle and myometrium (Figure 1h; Figure S4a–f). FOXO3 was also mainly upregulated in aged cornea, although upregulated in a few cells within other aging tissues, such as macrophage from aging skeletal muscle and fibroblast in aging myometrium (Figure S4g). Further, we examined the expression changes of other TFs known to regulate antioxidant gene expression, such as NRF2 (Ma, 2013) and YAP1 (Shao et al., 2014). Although these genes were upregulated in aged corneal cells, they exhibited distinct expression patterns during aging in other organ, underscoring the specific roles of ZNF281 and FOXO3 in corneal aging (Figures S3f,g and S4h,i).

We then analyzed the expression of ZNF281 targeted genes in stem cells from various organs, including monkey corneal cells (LSCs and TACs), skin stem cells (basal cells, BCs), hematopoietic stem cells (HSCs), MSCs, and neural stem cells (NSCs) from the hippocampus, and mesenchymal stem cells (MSCs) (Leveque et al., 2019), and found that the expression of these genes increased in LSCs, with minor changes in BCs, HSCs, and NSCs during aging. In contrast, the expression of some of these genes even decreased in aging MSCs compared to the young MSCs (Figure 1i). Moreover, we compared the expression levels of antioxidant genes during the aging and observed specific upregulation in LSCs and TACs. The expression of these genes was downregulated in human MSCs, consistent with the changes in ZNF281 and FOXO3 expression levels (Figure 1j **)**.

To further explore the role of ZNF281 and FOXO3, we analyzed their expression changes in other corneal diseases. We investigated the role of ZNF281 and FOXO3 using single‐cell transcriptomic datasets from human keratoconus corneas (Dou et al., 2022), a disease characterized by decreased antioxidant capacity. We found downregulation of ZNF281 and FOXO3, along with most of their targeted antioxidant genes (Figure S5) in keratoconus corneas, suggesting that the dysregulation of the antioxidant programs mediated by ZNF281 and FOXO3 may be involved in the development of keratoconus.

In summary, our results showed that ZNF281 and FOXO3 were specifically upregulated in aged corneal epithelial cells, indicating their crucial role in maintaining corneal homeostasis during aging.

### 2.3 ZNF281 exhibits anti‐aging capabilities

**FIGURE 2:**
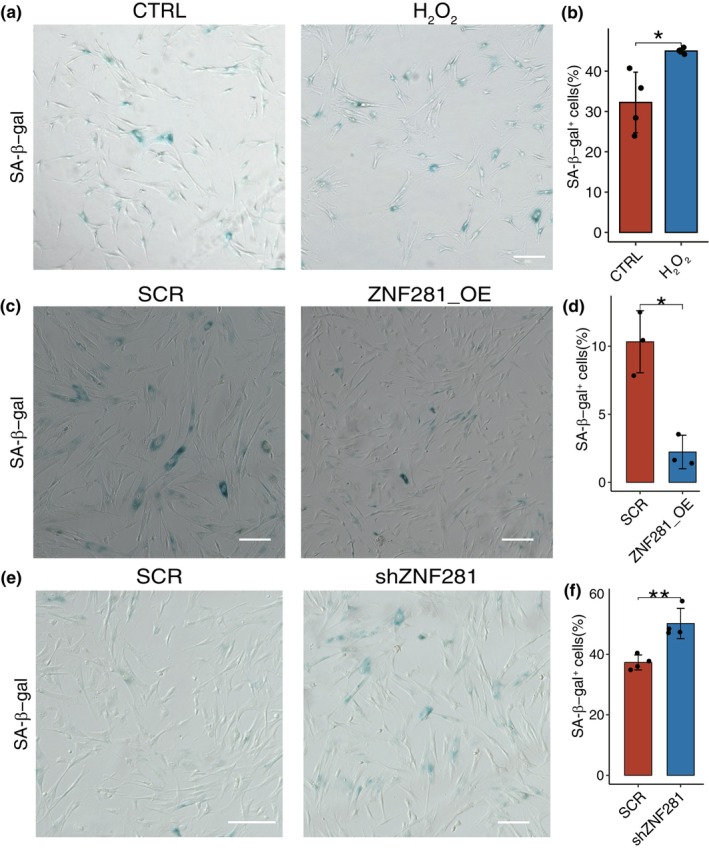
The antiaging role of ZNF281 in MSC. (a) Representative micrographs showing SA‐β‐gal‐positive cells among control MSCs and H_2_O_2_‐treated MSCs, Scale bars = 20 μm. (b) Bar plots showing the increase in SA‐β‐gal‐positive MSCs number following H_2_O_2_ treatment. Data are presented as the mean ± SEM. *n* = 4 for each group. *p < 0.05. (c) Representative micrographs showing SA‐β‐gal‐positive (aging) cells among control MSCs and *ZNF281*‐overexpressing MSCs. (d) Bar plots showing the decrease in SA‐β‐gal‐positive MSCs when *ZNF281* is overexpressed. Data are presented as the mean ± SEM. *n* = 3 for each group. *p < 0.05. (e) Representative micrographs showing SA‐β‐gal‐positive (aging) cells among control MSCs and *ZNF281*‐knockdown MSCs. (f) Bar plots showing the increase in SA‐β‐gal‐positive MSCs when *ZNF281* is knocked down. Data are presented as the mean ± SEM. *n* = 4 for each group. **p < 0.01.

To provide further evidence that the ZNF281 and FOXO3 can protect against aging‐associated oxidative damage and aging, we assessed whether manipulation of their expression levels could modulate these processes in human MSCs, which exhibit *ZNF281* downregulation during normal aging (Figure 1j). To reveal the role of oxidant stress in cellular aging, we initially treated MSCs with H₂O₂ to induce oxidative stress and found an increased proportion of β‐gal‐positive cells, a characteristic marker of cellular senescence (Figure 2a,b). We found that overexpressing ZNF281 led to a decrease in the number of SA‐β‐gal‐positive MSCs (Figure 2c,d; Figure S6a **)**, while knocking down ZNF281 resulted in an increase in the number of SA‐β‐gal‐positive MSCs, indicating an overall anti‐senescence effect of ZNF281 (Figure 2e,f; Figure S6b). Furthermore, knockdown of ZNF281 in human corneal epithelial cells (CECs), human fibroblast (HFFs), and retinal pigment epithelial (RPEs) results in significantly increased SA‐β‐gal‐positive cells, and knockdown of FOXO3 also increased SA‐β‐gal‐positive cells (Figure S6c–i). In summary, our results suggesting that ZNF281 plays a role in conferring resistance to senescence, possibly through enhancing antioxidant capacity.

In conclusion, our results underscore the pathophysiological importance of ZNF281 and FOXO3 autologous upregulation during corneal epithelial aging. We also hypothesize that the activation of ZNF281 and FOXO3 may enhance the antioxidant capacity of other stem cells to maintain tissue homeostasis and resist aging **(**Figure 1g **)**.

### 2.4 ZNF281 and FOXO3 exhibit a consistent ability to sense and mitigate oxidant stress

**FIGURE 3:**
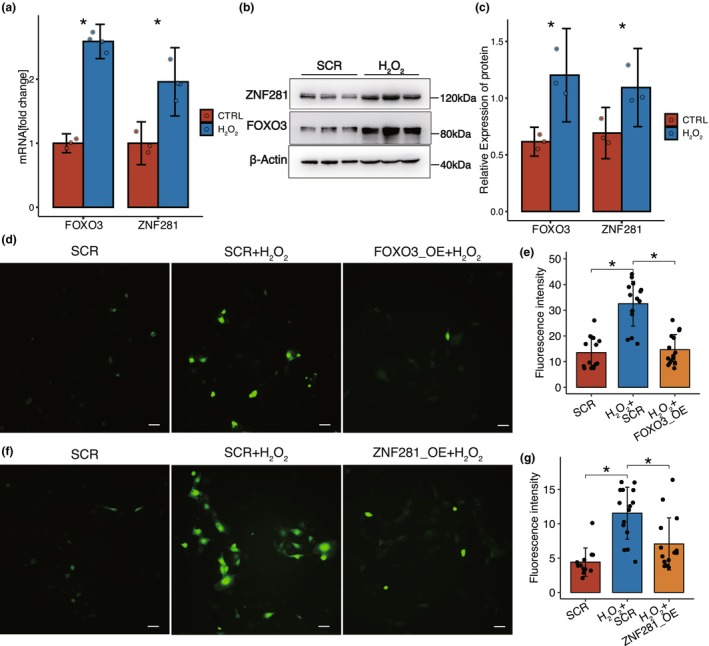
ZNF281 and FOXO3 exhibit a consistent ability to sense and mitigate oxidant stress. (a) Bar plot showing the *ZNF281* and *FOXO3* mRNA levels are upregulated by H_2_O_2_ treatment in hCECs. Data are presented as the mean ± SEM. *n* = 3 for CTRL and *n* = 4 for H_2_O_2_ group. *p < 0.05. (b) Sample Western blot revealing upregulation of ZNF281 and FOXO3 by ROS accumulation. (c) Bar plot quantifying the Western blot reveals a significant increase in ZNF281 and FOXO3 protein levels as a result of ROS elevation. Data are presented as the mean ± SEM. *n* = 3 for each group. *p < 0.05. (d) Representative micrographs showing ROS levels as measured by DCFH‐DA fluorescence in control hCECs, *FOXO3*‐overexpressing (OE) hCECs, scramble control H_2_O_2_‐treated hCECs, and *FOXO3*‐overexpressing H_2_O_2_‐treated hCECs, Scale bars = 20 μm. (e) Bar plot of mean fluorescence intensity in (d). Data are presented as the mean ± SEM. *n* = 15 for each group. *p < 0.05. f, Representative micrographs showing ROS levels as measured by DCFH‐DA fluorescence in control hCECs, *ZNF281*‐overexpressing hCECs, scramble control H_2_O_2_‐treated hCECs, and *ZNF281*‐overexpressing H_2_O_2_‐treated hCECs, Scale bars = 20 μm. (g) Bar plot of mean fluorescence intensity in (f). Data are presented as the mean ± SEM. *n* = 15 for each group. *p < 0.05.

To further examine whether *ZNF281* and *FOXO3* mediate antioxidant capacity of epithelial cells, we first examined expression in human CECs treated with H_2_O_2_ (Figure S7a,b) and found that ZNF281 and FOXO3 were upregulated at both mRNA and protein levels following exposure (Figure 3a–c; Figure S8), indicating that ZNF281 and FOXO3 can sense the elevated ROS levels. Furthermore, we conducted additional investigations to assess the functions of ZNF281 and FOXO3. By knocking down FOXO3, we found an elevation in ROS levels (Figure S7c,d) suggesting that FOXO3 plays a role in diminishing oxidative stress. Additionally, DCFH‐DA staining for cellular ROS levels demonstrated that overexpression of either ZNF281 or FOXO3 decreased H_2_O_2_‐induced ROS (Figure 3d–g), implying their role in enhancing cellular antioxidant capacity. Overall, these findings confirm that ZNF281 and FOXO3 have the ability to activate autologously, allowing them to sense and mitigate oxidative stress. In doing so, they contribute to the maintenance of redox homeostasis in aged corneal epithelial cells. Taken together, these results provide further support for the idea that ZNF281 and FOXO3 are involved in protecting against aging‐related oxidative damage and senescence in corneal epithelium and MSCs.

### 2.5 ZNF281 and FOXO3 form a positive feedback loop to collaboratively mitigate oxidative stress collaboratively

**FIGURE 4:**
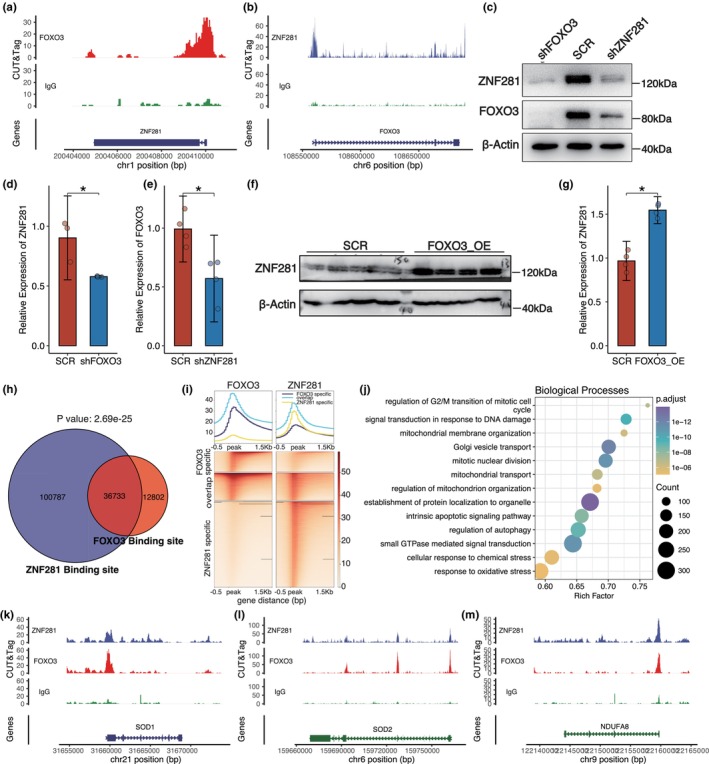
ZNF281 and FOXO3 form a positive feedback loop to mitigate oxidative stress collaboratively. (a, b) Track plot showing the binding of ZNF281 to the *FOXO3* promoter region (a) and vice versa (b). (c) Sample Western blot showing significant reductions in ZNF281 and FOXO3 protein expression levels upon *FOXO3* or *ZNF281* knockdown, respectively. (d, e) Bar plots of Western blotting results showing that ZNF281 protein levels were significantly reduced by FOXO3 knockdown (d) and FOXO3 protein levels by ZNF281 knockdown (e). (d) Data are presented as the mean ± SEM. *n* = 3 for each group. *p < 0.05. (e) Data are presented as the mean ± SEM. *n* = 4 for each group. *p < 0.05. (f, g) Sample Western blot (f) and densitometric quantitation (g) revealing that *FOXO3* overexpression significantly enhanced ZNF281 protein level. Data are presented as the mean ± SEM. *n* = 3 for each group. *p < 0.05. (h) Venn plot showing that the majority of whole‐genome FOXO3 binding sites are co‐occupied by ZNF281. (i) Heatmaps depicting the CUT&Tag intensities of ZNF281 and FOXO3 based on FOXO3‐specific peaks, ZNF281‐specific peaks, and overlapping peaks, respectively. (j) Bubble plot of enriched GO terms for genes nearest to the overlapping peaks of ZNF281 and FOXO3. (k–m) Track plots showing the binding of ZNF281 and FOXO3 to the promoter regions of antioxidant genes (k, l) and genes involved in the OXPHOS (m).

Through co‐expression analysis, we found that the expression of ZNF281 and FOXO3 was positive correlated in corneal epithelial cell (Figure S9a). To assess potential interactions between ZNF281 and FOXO3, we performed CUT&Tag experiments on human CECs (Figure S9b). Our results suggested that ZNF281 can bind to the FOXO3 promoter and vice versa (Figure 4a,b), suggesting mutual regulation of expression. Indeed, knockdown and overexpression assays, coupled with qPCR, revealed that ZNF281 and FOXO3 are mutually regulated; specifically, ZNF281 knockdown reduces FOXO3 expression (Figure S9c), while its overexpression enhances FOXO3 expression (Figure S9d), and similarly, FOXO3 manipulation affects ZNF281 levels (Figure S9e–g). Protein expression levels of both ZNF281 and FOXO3 also decreased following the knockdown of *ZNF281* and *FOXO3* (Figure 4c–e; Figures S9h,i and S10a), and overexpression of FOXO3 elevated ZNF281 protein levels (Figure 4f,g; Figure S10b). Collectively, these findings indicate that ZNF281 and FOXO3 form a positive feedback loop, mutually enhancing expression and thereby coordinating the expression of target gene networks.

Furthermore, we found a significant overlap of genome‐wide binding sites by ZNF281 and FOXO3 respectively (Figure 4h). Specifically, there were 35,755 binding sites shared by ZNF281 and FOXO3 (Figure 4h,i), and the corresponding genes were enriched in functions like mitotic nuclear division, and response to oxidative stress (Figure 4j). We observed the promoters of SODs, such as SOD1 and SOD2, were co‐occupied by ZNF281 and FOXO3 in the CECs (Figure 4k,l; Figure S11 **)**, which were consistently upregulated in aged corneal epithelium (Figure 1i,j). In addition, we found that the ZNF281 and FOXO3 bind to the promoter regions of genes implicated in oxidative phosphorylation (OXPHOS), such as *NDUFA8*, *UQCR10*, *UQCRH* (Figure 4m). In summary, our findings indicate that ZNF281 and FOXO3 collaborate to activate SODs and inhibit genes related to OXPHOS, thus helping to mitigate oxidative stress.

To further analysis the cell–cell interaction in aging cornea, we then performed cell communication analysis in aging corneal cells. This analysis revealed enhanced communication strength in aging corneal epithelial cells, with 29 pathways significantly altered during the aging process (Figure S12a, b **)**. We found that the EGF pathways were enhanced in cells with upregulated ZNF281 and FOXO3 expression during aging (Figure S12c–e). Ligands AREG and HBEGF, both target genes of ZNF281 and FOXO3 (Figure S12f, g), and their receptor EGFR were found to act as major signaling molecules from cells significantly expressing ZNF281 and FOXO3, potentially influence their proliferation and migration. Together, these analyses revealed that ZNF281 and FOXO3 could regulate cell–cell communication during corneal epithelial cell aging processes.

### 2.6 ZNF281 and FOXO3 both regulate OXPHOS in the corneal epithelium

**FIGURE 5:**
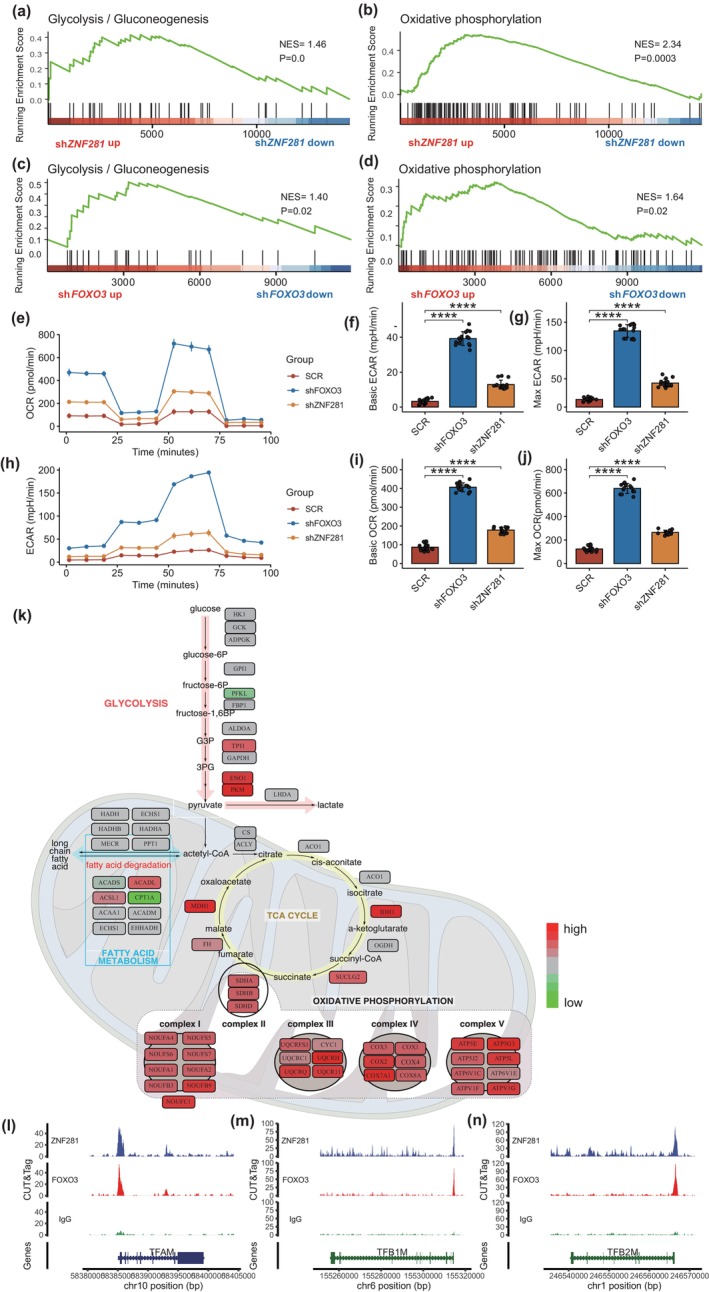
ZNF281 and FOXO3 both regulate oxidative phosphorylation in the corneal epithelium. (a–d) Enrichment plots showing upregulation of the Glycolysis/Gluconeogenesis gene sets by *ZNF281* knockdown (a), upregulation of the “Oxidative Phosphorylation (OXPHOS)”gene set by *ZNF281* knockdown (b), upregulation of the“Glycolysis/Gluconeogenesis”gene set by *FOXO3* knockdown (c), and upregulation of the OXPHOS gene set by *FOXO3* knockdown (d). (e) Seahorse XF analysis of extracellular acidification rate (ECAR) in hCECs following ZNF281 and FOXO3 knockdown. (f, g) Bar plots showing the significantly upregulation of glycolysis rate (f) and glycolytic capacity (g) after *ZNF281* and *FOXO3* knockdown. (f) Data are presented as the mean ± SEM. *n* = 15 for each group. ****p < 0.0001. (g) Data are presented as the mean ± SEM. *n* = 15 for each group. ****p < 0.0001. (h) Seahorse XF analysis of oxygen consumption rate (OCR) in hCECs following ZNF281 and FOXO3 knockdown. (i, j) Bar plots showing the significantly upregulation of basal OCR (i) and max OCR (j) following ZNF281 and FOXO3 knockdown. (i) Data are presented as the mean ± SEM. *n* = 15 for each group. ****p < 0.0001. (j) Data are presented as the mean ± SEM. *n* = 15 for each group. ****p < 0.0001. (k) Heatmap presents changes in metabolism‐related gene expression levels after *ZNF281* knockdown. (l–n) Track plots showing the binding of ZNF281 and FOXO3 to the promoter regions of TFAM (l), TFB1M (m), and TFB2M (n).

Through gene set enrichment analysis, we observed that the expression levels of ZNF281 and FOXO3 significantly impact the regulation of glycolysis and OXPHOS pathways in corneal epithelial cells (Figure 5a–d; Figure S13). We investigated the potential effects of ZNF281 and FOXO3 on cellular metabolic profiles by examining their influence on OXPHOS, a major source of ROS within the cell. The knockdown of *ZNF281* or *FOXO3* significantly increased the extracellular acidification rate (ECAR) as measured using a Seahorse extracellular flux analyzer, consistent with enhanced glycolysis and glycolysis capacity (Figure 5e–g). Similarly, *ZNF281* or *FOXO3* knockdown increased oxygen consumption rate (OCR), indicating enhanced OXPHOS (Figure 5h–j). Furthermore, the knockdown of *ZNF281* or *FOXO3* significantly increased the expression of genes in the OXPHOS pathway (Figure 5k; Figure S14). Subsequently, we further performed analyses to evaluate the impact of ZNF281 and FOXO3 on mitochondrial biogenesis. We found that both ZNF281 and FOXO3 can bind to the promoter TFAM, TFB1M, and TFB2M, genes regulating mitochondrial biogenesis (Figure 5l–n). Our data suggest that ZNF281 and FOXO3 not only reduce glycolysis and OXPHOS activities but also influence mitochondrial number. In summary, these results collectively demonstrate that ZNF281 and FOXO3 negatively influence glycolysis and OXPHOS activities and reduce the number of mitochondrial numbers in the corneal epithelium.

## 3 DISCUSSION

**FIGURE 6:**
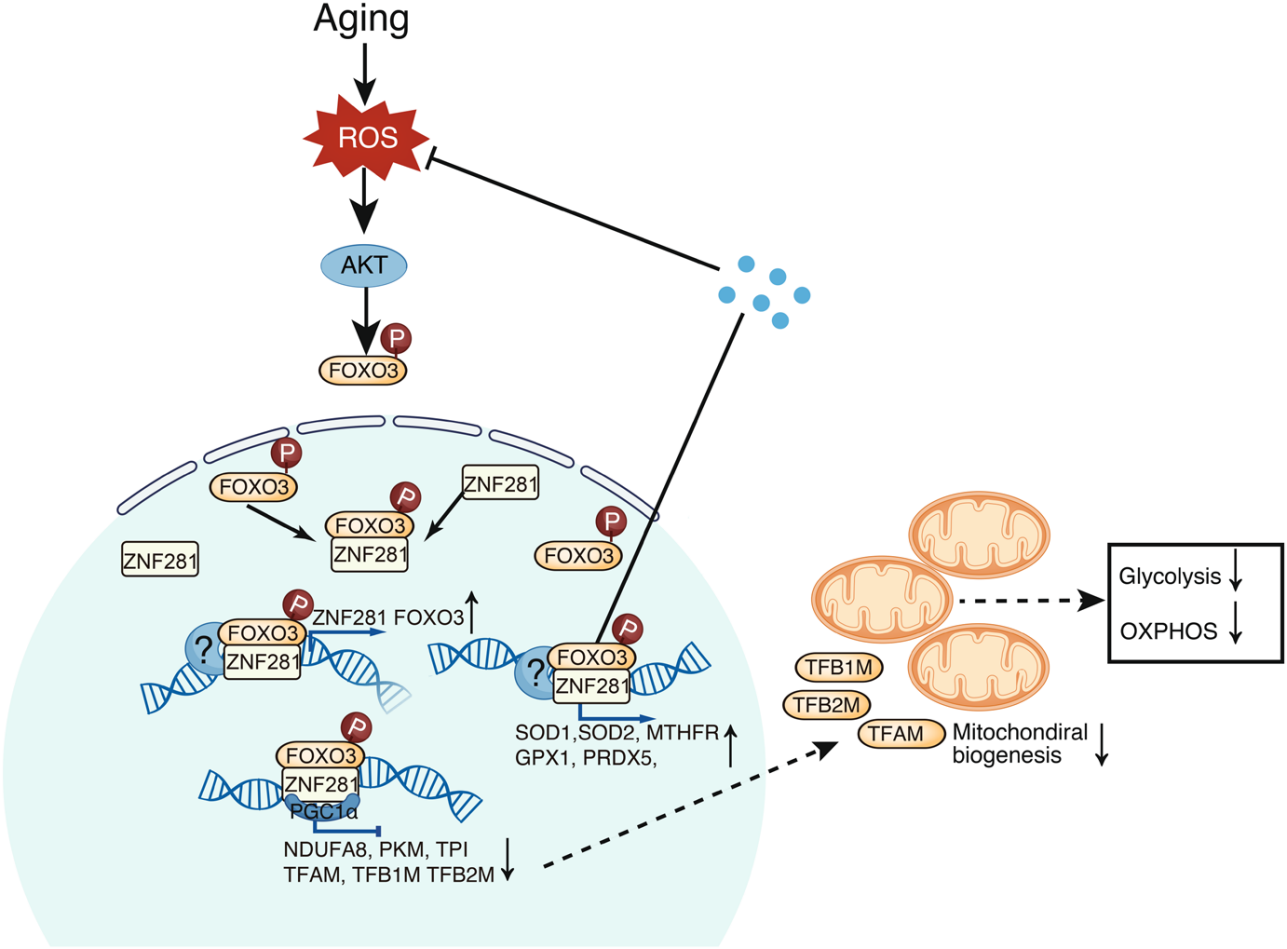
The diagram illustrates the functions of ZNF281 and FOXO3 in corneal epithelial cells during aging. As cells age, there is an increase in the production of reactive oxygen species (ROS), leading to oxidative stress. ZNF281 and FOXO3 serve as sensors for ROS and mutually enhance their expression levels. A potential mechanism for ROS‐mediated upregulation of ZNF281 and FOXO3 involves the induction of FOXO3 phosphorylation by ROS. This phosphorylation prompts FOXO3's translocation from the cytoplasm to the nucleus. Once inside the nucleus, FOXO3 and ZNF281 form a complex with other co‐regulators to upregulate their own expression. The elevated expression of these transcription factors subsequently upregulates antioxidant genes expression, such as SOD1 and SOD2, while downregulating genes related to OXPHOS in mitochondria, and those that govern mitochondrial biogenesis. This coordinated response effectively reduces cellular ROS levels.

In this study, we created a comprehensive single‐cell transcriptomic atlas of the corneal epithelium in both young and aged monkeys. By performing comparative single‐cell transcriptomic analysis of the aged cornea and other aged organs, we discovered the specific activation of a ZNF281‐coding program in the cornea. Notably, we identified a transcriptional positive‐feedback loop between the TF ZNF281 and FOXO3, which enhances ROS sensing and stimulates antioxidant responses in the majority of corneal epithelium. Furthermore, this ZNF281‐FOXO3 loop suppresses mitochondrial metabolism, the primary source of intracellular ROS, and upregulates antioxidative genes like SODs. We hypothesize that the continuous activity of the ZNF281‐FOXO3 loop plays a crucial role in the observed resilience of the corneal epithelium against age‐related damage and aging. (Figure 6).

Our research has provided novel insights into the functions and relationship of ZNF281 and FOXO3, which formed a positive feedback loop to mitigate oxidative stress collaboratively. While previous studies have shown that FOXO3 is often downregulated with aging in various cell types, such as arterial endothelial cells, leading to reduced efficiency in preventing and repairing DNA damage (Morris et al., 2015; Sanese et al., 2019; Yan et al., 2019; Zhang et al., 2020), our study presents an interesting contrast. We found that the corneal epithelium naturally maintains high levels of FOXO3 and ZNF281 expression as it ages, which helps to suppress ROS. Moreover, our findings indicate that the ZNF281‐FOXO3 loop not only senses and counteracts ROS accumulation, but also regulates the expression of antioxidant genes, such as SOD2 and SOD1, thus aiding in cellular ROS removal. Given that the knockdown of ZNF281 and FOXO3 in CECs, RPEs, and HFFs leads to cellular aging, we hypothesize that sustaining the activity of the ZNF281‐FOXO3 loop—either through its natural activation or through ectopic expression of ZNF281—could be an effective strategy to enhance resistance to aging.

Additionally, our results indicated that ZNF281 and FOXO3 not only reduce glycolysis and OXPHOS activities but also influence mitochondrial quantity. We observed that ZNF281 and FOXO3 bind concurrently to the promoter regions of genes involved in the glycolysis and OXPHOS pathways, thereby suppressing their expression and further diminishing ROS generation. Furthermore, our CUT&Tag results revealed that both ZNF281 and FOXO3 bind to the promoter regions of TFAM, TFB1M, and TFB2M, which are essential for mitochondrial biogenesis (Gleyzer et al., 2005), suggesting that ZNF281 and FOXO3 also contribute to the reduction in mitochondrial numbers. Consistent with our findings, a recent study has validated the role of ZNF281 in suppressing mitochondrial quantity (Zhao et al., 2023). Collectively, our results elucidate a novel mechanism through which ZNF281 and FOXO3 reduce cellular ROS levels. This expands our understanding of ZNF281's functions and sheds light on the intrinsic mechanisms involved in maintaining ROS homeostasis in the cornea.

There are several limitations to our study that should be acknowledged. Firstly, although we observed the antiaging functions of the ZNF281‐FOXO3 feedback loop in MSCs culture, these findings have not been validated in an in vivo environment. It is important to conduct further investigations to confirm these functions in animal models. Secondly, the sample size in our study was limited, and therefore, it would be beneficial to expand the sample size in future studies. Additionally, conducting a more comprehensive analysis using single‐cell atlases of aging tissues throughout the body could provide a stronger foundation for developing therapeutic approaches to combat aging and extend healthy lifespan.

## 4 METHODS

### 4.1 Animals

Two macaque monkeys (*Macaca fascicularis*), one 19‐year‐old (approximately equivalent to 60‐year‐old human) and one 4‐year‐old (comparable to 12‐year‐old human) healthy animal, were provided by Huazhen Laboratory Animal Breeding Centre (Guangzhou, China), an accredited primate research center in Guangzhou. All animal procedures were performed according to Principles for the Ethical Treatment of NonHuman Primates as well as the Statement for the Use of Animals in Ophthalmic and Vision Research and were approved by the Institutional Animal Care and Use Committee (IACUC) of Zhongshan Ophthalmic Center of Sun Yat‐sen University (2019–150). Animals were maintained at 25°C under a 12 h/12 h light/dark schedule at Huazhen Laboratory Animal Breeding Centre, and fed a commercial diet twice daily plus fruits once daily and were sufficient water.

### 4.2 Corneal epithelium dissociation and cell isolation

The cornea was excised under anesthesia and digested in a solution of Dispase II (10 mg/mL) at 37°C for 2 h. Subsequently, the corneal epithelium was carefully dissected using forceps, and transferred to a 1.5 mL EP tube containing Dispase II (2.5 mg/mL) at 37°C for 15 min with gentle shaking every 5 min to dissociate the cell aggregates into single cells. Single cell suspensions were diluted to about 1000 cells/μL in Ames Medium supplemented with 0.04% bovine serum albumin (BSA).

### 4.3 Cell culture

Human MSCs were cultured in DMEM medium supplemented with 15% fetal bovine serum, 0.1 mM nonessential amino acids, and 1% penicillin/streptomycin. Immortalized human corneal epithelial cells (hCECs) were cultured in DMEM‐F12 supplemented with 5 μg/mL human insulin (MCE), 10 ng/mL recombinant human epidermal growth factor (EGF, Sigma) and 1% streptomycin at 37°C under a 5% CO_2_ atmosphere. RPEs and HFFs were cultured in DMEM medium supplemented with 10% fetal bovine serum, and 1% penicillin/streptomycin. Cells were passaged and harvested for seeding using 0.05% Trypsin—EDTA.

### 4.4 Knockdown or overexpression of ZNF281 and FOXO3

Lentiviral vectors carrying shRNA against human FOXO3 and ZNF281, as well as FOXO3 and ZNF281 overexpression constructs, were generated and packaged by VectorBuilder (Guangzhou, China). For transfection, cell lines were seeded overnight and then incubated for 11 h in culture medium containing lentivirus at a multiplicity of infection (MOI) of 10 in the presence of 10 μg/mL polybrene. Following incubation, the lentivirus‐containing medium was replaced with fresh culture medium.

### 4.5 Real‐time quantitative PCR (RT‐qPCR)

Cells were collected by centrifugation and total RNA isolated using TRIzol Reagent (ThermoFisher Scientific) according to the manufacturer's instructions. A 2 μg sample of total RNA was reverse transcribed into cDNA using Reverse Transcription Master Mix (Tiangen). RT‐qPCR reactions were performed on a LightCycler® 480 System (Roche) using AceQ qPCR SYBR Green Master Mix (Vazyme) according to the manufacturer's protocol. Target gene expression was normalized to the expression of *ACTB*.

### 4.6 Western blotting

Cells were lysed in cold RIPA buffer and total protein measured using a BCA kit. Equal amounts of protein were separated by SDS‐PAGE and transferred to 0.2 mm PVDF membranes (Millipore). Membranes were incubated with primary antibodies at 4°C overnight and then with horseradish peroxidase (HRP)‐conjugated secondary antibodies. Grayscale images of membranes were analyzed using ImageJ.

### 4.7 Metabolic flux assays

ECAR and OCR were measured using the Seahorse XF24 system (Agilent Technologies). Briefly, 4.5 × 10^4^ cells were seeded with normal growth medium (DMEM‐F12, 5 μg/mL human insulin, 10 ng/mL recombinant human EGF, and 1% streptomycin) on Seahorse XF24 Cell Culture Microplates. After 24 h, cells were washed with 900 μL XF Assay Medium (pH 7.4) supplemented with 2 mM L‐glutamine, with or without 10 mM glucose, and with or without 1 mM Na pyruvate. Growth medium was then exchanged with 500 μL XF Assay Medium. Cells were placed in a CO_2_‐free incubator at 37°C for 1 h, followed by measurement of ECAR and OCR using the Seahorse XF Glycolysis Stress Test Kit (Agilent Technologies) and Seahorse XF Cell Mito Stress Test Kit (Agilent Technologies), respectively, according to the manufacturer's protocols. Subsequent, the cell count was accurately determined using hormony to ensure precise normalization of the ECAR and OCR measurements, facilitating reliable and consistent data interpretation.

### 4.8 Measurement of ROS

Intracellular ROS levels were measured using the fluorescent probe 2′, 7′‐dichlorodihydrofluorescein diacetate (DCFH‐DA, Beyotime, China). Cells were seeded onto 96‐well plates at 5 × 10^4^ cells/well, incubated with 10 μM DCFH‐DA for 20 min at 37°C in the dark, and washed three times in serum‐free medium. Fluorescence emission intensity at 488 nm was calculated using ImageJ. Three biological replicates were conducted for each treatment.

### 4.9 Senescence‐associated β‐galactosidase staining

For senescence‐associated β‐galactosidase (SA‐β‐gal) staining, cells were first washed with PBS, fixed in 2% formaldehyde plus 0.2% glutaraldehyde for 5 min, and then incubated overnight in freshly prepared staining solution at 37°C. Staining intensity was analyzed using ImageJ.

### 4.10 Single‐cell RNA‐seq library preparation and sequencing

Single‐cell RNA‐seq libraries were prepared using the Single‐cell 3' Library and Gel Bead Kit V2 and Gel Bead Kit (both from 10x Genomics, Pleasanton, CA, USA) according to the manufacturer's protocol. Briefly, single epithelial cells were lysed and the released RNA subjected to barcoded reverse transcription in gel beads in emulsion (GEMs). Libraries were sequenced on an Illumina HiSeq X Ten system.

### 4.11 Bulk RNA‐seq libraries preparation and sequencing

*FOXO3* knockdown human corneal epithelial cells (KD‐hCECs) and scrambled control hCECs were prepared as described and harvested. Total RNA was obtained using TRIzol Reagent (ThermoFisher Scientific) and RNA quality was confirmed using a Bioanalyzer 2100 system (Agilent Technologies). Sequencing libraries were generated using the NEBNext Ultra™ RNA Library Prep Kit (NEB) and sequenced on the Illumina HiSeq X Ten system. We set 2 biological replicates for each group to eliminate technical variance.

### 4.12 Cleavage under targets and tagmentation (CUT&Tag) sequencing

CUT&Tag sequencing was performed strictly following the kit manufacturer's protocol (Vazyme, S602‐01). To isolate cell nuclei, cells were first immersed in cooled NE1 buffer for 10 min, centrifuged, rehydrated in wash buffer, and weakly cross‐linked in 16% formaldehyde for 2 min. The cross‐linking reaction was terminated by the addition of 2.5 M glycine. The isolated nuclei were magnetically tagged with ConA beads, immersed in antibody‐binding buffer, and divided into several 0.5‐mL tubes for antibody incubation overnight at room temperature or 4°C. Postincubation, samples were washed to eliminate nonaffixed primary antibody, rehydrated, incubated in wash buffer supplemented with the secondary antibody at 4°C for one hour, washed again, resuscitated in the 300‐wash buffer inclusive of pA‐Tn5, and incubated at 4°C for another hour. Samples were washed in 300‐wash, replenished in tagmentation buffer, and maintained at 37°C for 1 h to conduct the Tn5 tagmentation reaction. Samples were then washed using TAPS wash buffer supplemented with 5 μL release solution and incubated in a thermocycler at 58°C for one hour to extract Tn5 and ready the tagmented chromatin for PCR. A neutralizing solution was added, followed by the introduction of barcoded primers and NEBNext PCR mix. Finally, PCR amplification was conducted using 12–14 swift cycles, and samples were cleaned by a single round of SPRIselect bead incubation. Yields were quantified using a TapeStation Bioanalyzer instrument and compiled for sequencing.

### 4.13 Processing raw data from scRNA‐seq

Single‐cell RNA‐seq data were processed using CellRanger (Version 3.1.0). Briefly, the FASTQ files were mapped to the *Macaca fascicularis* genome (Macaca_fascicularis_5.0) and used to generate UMI‐based gene–barcode matrices. The genome annotation was based on NCBI *Macaca fascicularis* Annotation Release 98. Gene–barcode matrices were further analyzed using Seurat (Stuart et al., 2019) (version 4.1.0) following the tutorial. Cells with more than 500 detectable genes were retained for subsequent analysis.

### 4.14 Cell type identification

We used the CCA of Seurat to integrate the two datasets from healthy and aging monkeys. The top 2000 highly variable genes from the two datasets were selected, normalized, and integrated. Principal component analysis (PCA) was then conducted on the integrated data and the significant principal components (*p* < 10^−5^) were selected. Clustering was then performed using the *FindClusters* function. Cluster‐specific genes were identified using the *FindAllMarker* function and compared to well‐known markers of corneal epithelium cell types for annotation of each cluster.

### 4.15 Identification of DEGs from scRNA‐seq data

We performed differential gene expression analysis of the aging dataset versus the young dataset using the *FindMarkers* function of Seurat. Only genes with adjusted *p* < 0.05 and absolute logarithmic2 fold‐change >0.25 were selected as DEGs.

### 4.16 Analysis of publicly available scRNA‐seq datasets

Raw scRNA‐seq datasets from aging monkey tissues, including lung, heart, and hippocampus, were downloaded from the Genome Sequence Archive (GSA) and processed as described for the corneal scRNA‐seq dataset. The old and young datasets of aging bone, and myometrium were downloaded from the GEO and processed as described for the corneal scRNA‐seq dataset. The processed scRNA‐seq dataset of skeletal muscle was downloaded from https://www.muscleageingcellatlas.org, while the aging brain dataset was downloaded from https://doi.org/10.7303/SYN51111084.1. To compare the differences in ZNF281 and FOXO3 expression across different tissues during the aging process, we performed differential gene expression analysis between aging and young groups within the same dataset for each tissue. The fold change of each gene was used for comparisons across different tissues and species.

### 4.17 Enrichment analysis and gene set score analysis

Enrichment analysis of DEGs was performed using the *enricher* function of the clusterProfiler (Yu et al., 2012) (version 3.14.3) R package, and further gene set enrichment analysis was performed using the *GSEA* function of clusterProfiler. Gene sets were obtained using the msigdbr (Liberzon et al., 2015) (version 7.2.1) R package, and the Seurat *AddModuleScore* function was used to calculate gene sets scores for estimation of relative gene expression levels within pathways in single cells.

### 4.18 Transcriptional regulatory network analysis

Transcriptional regulatory networks were constructed using SCENIC (Aibar et al., 2017) (version 1.1.2–2) with default parameters. hg38 TFs database was download using RcisTarget as reference. Gene regulatory networks were inferred with GENIE3 using all detected genes in nonhuman primate cornea. Enriched TF‐binding motifs, predicted candidate target genes (regulons), and regulon activity were inferred by RcisTarget.

### 4.19 Analysis of data from bulk RNA‐seq

After adapter trimming and removal of low‐quality reads using Trim Galore (version 0.4.4), remaining reads were aligned to the human genome (hg38) using STAR (version 2.7.3a) (Dobin et al., 2013) in the pair‐end mode with default parameters. Genome annotation references were based on gencode release v30. Uniquely mapped reads were retained and counted by HTSeq (version 0.11.2) (Dobin et al., 2013) to generate a gene‐sample matrix. DEGs between *FOXO3*‐KD, *ZNF281*‐KD and scramble groups were identified using DESeq2 (version 1.26.0) (Love et al., 2014) with the criteria of absolute fold change >1.3 and false discovery rate (FDR) adjusted *p* < 0.05.

### 4.20 Cell–cell communication analysis

The R package CellChat (Jin et al., 2021) was employed for cell–cell communication analysis. Briefly, the normalized gene expression matrix and cell labels generated by Seurat were used as input for CellChat. Overexpressed ligands and receptors in each cell type were calculated and then projected onto the protein–protein interaction network. To infer biologically significant cell–cell communication, each interaction was assigned a probability value, and a permutation test was performed. Cell–cell communication alterations in the aging cornea were analyzed using joint manifold learning and quantitative contrasts of multiple cell–cell communication networks. Finally, communication networks were visualized using circle plots, and signaling pathways were illustrated using bubble plots.

### 4.21 CUT&Tag data processing

Paired‐end reads were aligned to UCSC hg38 using Bowtie2 (Langmead & Salzberg, 2012) version 2.3.4.3 with the following options: —end‐to‐end, —very‐sensitive, —no‐mixed, —no‐discordant‐q—phred33‐I 10‐X 700. Peaks were called using MACS3 (Zhang et al., 2008) with the following options:‐f BAMPE, ‐g hs, ‐B, ‐q 0.01. Co‐occupied binding sites were identified using the intersect function from the BEDTools suite. DeepTools was further applied for heatmap visualization with the computeMatrix and plotHeatmap functions. ChIPSeeker was used to annotate the nearest gene to each peak.

### 4.22 Statistical analyses

Date are presented as mean ± SEM. All experimental data were statistically analyzed using R software (R4.2). Comparisons were performed using the two‐tailed Student's t‐test. *p* values lower than 0.05 are considered statistically significant. *, **, *** and **** indicate *p* < 0.05, *p* < 0.01, *p* < 0.001 and *p* < 0.0001, respectively.

## AUTHOR CONTRIBUTIONS

Y.H. conceived and designed the study. X.C., Z.C., X.H., W.D., S.Z., J.C., and L.L. performed experiments. Y.H., Y.X., J.Z., and X.L. analyzed the data and performed statistical analyses. Y.H., Y.X., C.X., and L.L. interpreted the data and wrote the manuscript in discussion with all authors.

## CONFLICT OF INTEREST STATEMENT

The authors declare no competing interests.

## Supporting information


Data S1.

## Data Availability

All sequencing data generated in this study are available at Gene Expression Omnibus (GEO) under accession number GSE197745 (https://www.ncbi.nlm.nih.gov/geo/query/acc.cgi?acc=GSE197745). The aging skin scRNA‐seq data was downloaded from the GSA under accession number HRA000395. Aging hippocampal scRNA‐seq data was obtained from GSA under accession number CRA004080. Aging heart and lung scRNA‐seq data were downloaded from GSA under accession numbers CRA002689 and CRA002577, respectively. Aging bone marrow scRNA‐seq data was acquired from the National Center for Biotechnology Information (NCBI) GEO under accession number GSE120221, and aging MSC data was obtained from NCBI under the BioProject accession number PRJNA510912. The processed scRNA‐seq dataset of skeletal muscle was downloaded from https://www.muscleageingcellatlas.org, while the aging brain dataset was downloaded from https://doi.org/10.7303/SYN51111084.1. The aging myometrium dataset was obtained from GEO under accession number GSE236660. The aging human cornea dataset was acquired from GEO under accession number GSE218123, and the young human cornea dataset was obtained from GEO under accession number GSE153515.
