# Supplementary material for: Comparative single‐cell transcriptomic analysis across tissues of aging primates reveals specific autologous activation of ZNF281 to mitigate oxidative stress in cornea
Source: Aging Cell. 2024 Sep 10;23(12):e14319. doi: 10.1111/acel.14319 (PMC11634732; doi:10.1111/acel.14319)
Supplement: Supplementary file 1 — Data S1. [file ACEL-23-e14319-s001.docx]

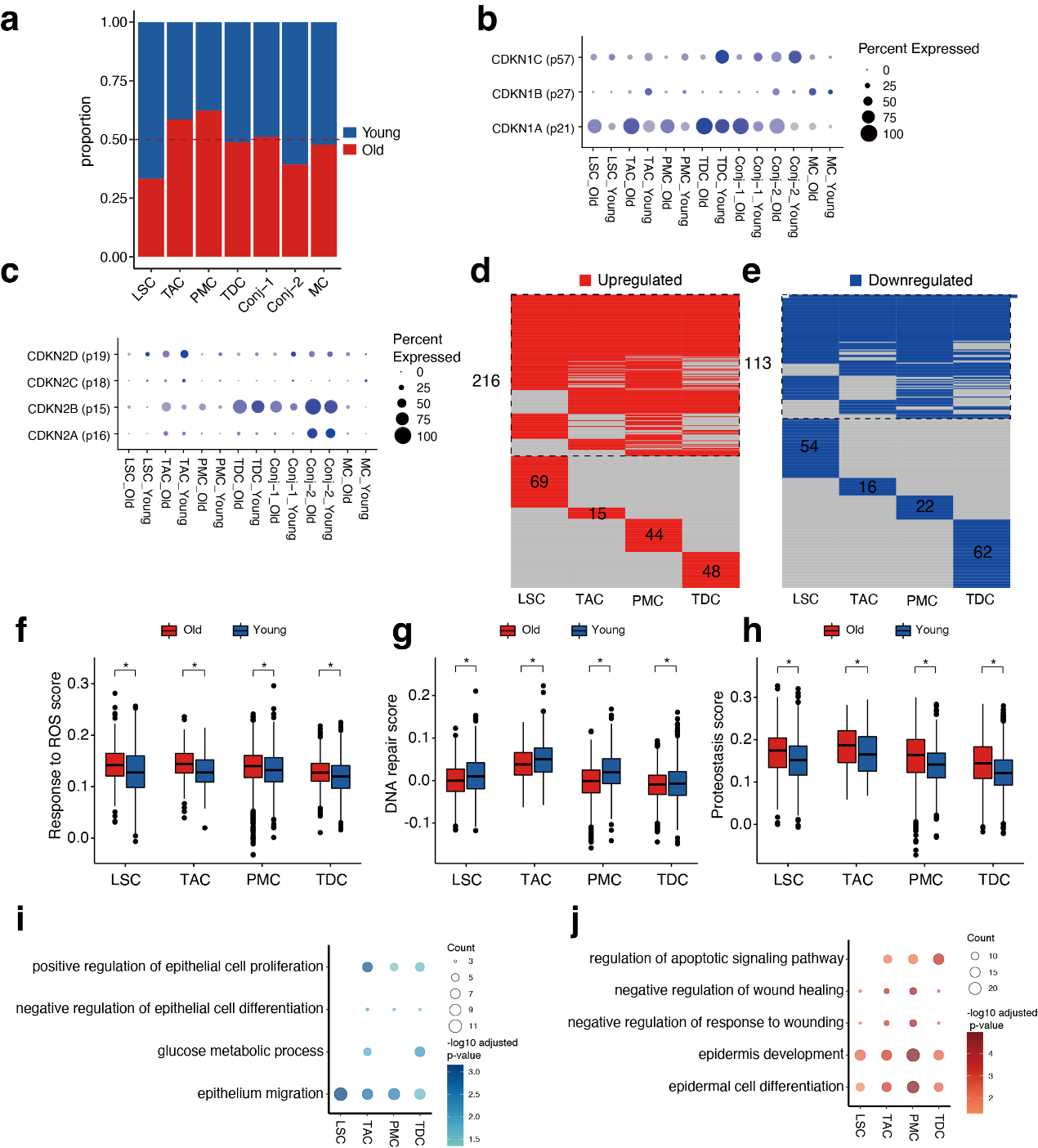


**Supplementary Fig. 1.** **Transcriptomic alteration during corneal epithelium aging. a,** Bar plots of the cell cycle stage distribution for each cell type in young and aged corneal epithelium. **b-c,** Dot plots showing changes in the expression levels of CDKN1A, CDKN1B, and CDKN1C (**b**) and of CDKN2A, CDKN2B, CDKN2C, and CDKN2D (**c**) during corneal epithelium aging. **d-e,** Heatmaps of upregulated **(d)** and downregulated **(e)** differentially expressed genes (DEGs) associated with aging for each cell type. **f-h,** Violin plots of gene set scores related to ‘Response to ROS’ (**f**), ‘DNA repair’ (**g**), and ‘Proteostasis’ (**h**) in young and old corneal epithelium. **i,** Dot plots showing shared GO terms of upregulated DEGs across cell types. **j,** Dot plots showing shared GO terms of downregulated DEGs across cell types.


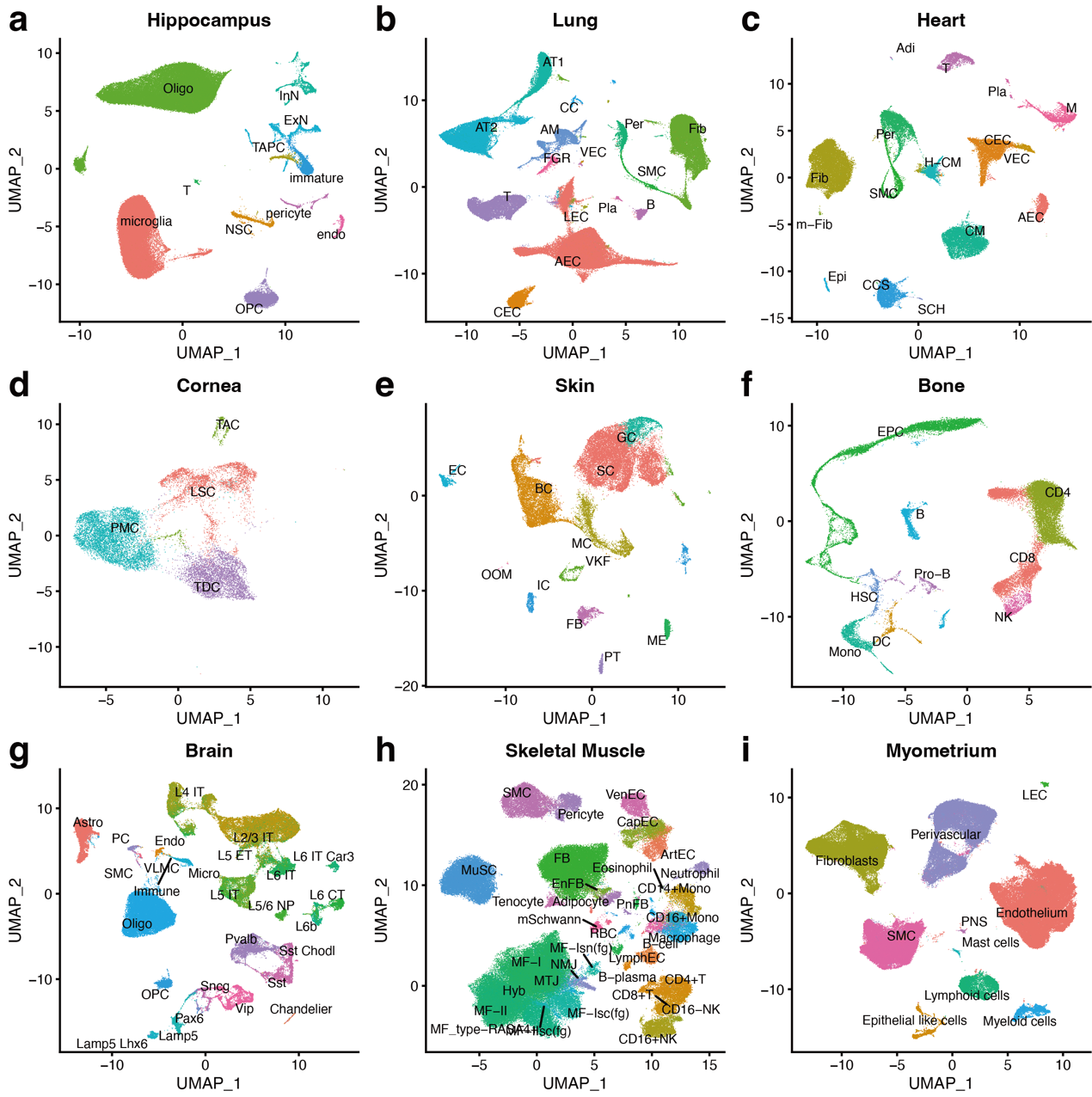


**Supplementary Fig. 2. UMAP visualization of all cell types in scRNA-seq of aging organs. a,** UMAP plot showing different cell types in the monkey hippocampus. NSC, neural stem cells; TAPC, transiently amplifying progenitor cells; ImN, immature neurons; ExN, excitatory neurons; InN, inhibitory neurons; OPC, oligodendrocyte progenitor cells; Oligo, oligodendrocytes; EC, endothelial cells; pericytes; VLMC, vascular leptomeningeal cells. **b,** UMAP plot showing different cell types in monkey lung. AEC, arterial endothelial cell; CEC, capillary endothelial cell; VEC, venous endothelial cell; LEC, lymphatic endothelial cell; Fib, fibroblast; SMC, smooth muscle cell; Per, pericyte; AT1, alveolar type I cell; AT2, alveolar type II cell; CC, ciliated cell; AM, alveolar macrophage; Pla, plasmocyte; FGR, FGR-positive cell. **c,** UMAP plot showing different cell types in monkey heart. M-Fib, myofibroblast; Fib, fibroblast; AEC, arterial endothelial cell; CEC, capillary endothelial cell; VEC, venous endothelial cell; CM, cardiomyocyte; H-CM, hypertrophic cardiomyocyte; Epi, epicardial cell; CCS, cardiac conduction system cell; Pla, plasmocyte; SCH, Schwann cell; Adi, adipose cell; SMC, smooth muscle cell; Per, pericyte; M, macrophage. **d,** UMAP plot showing different cell types in the cornea. LSC, limbal stem cell; TAC, transit amplifying cell; PMC, post-mitotic cell; TDC, terminally differentiated cell. **e**, UMAP plot showing different cell types in the human skin. MC, mitotic cell; PT, pericyte; OOM, orbicularis oculi muscle; BC, basal cell; VHF, vellus hair follicle; ME, melanocyte; SC, spinous cell; GC, granular cell; EC, endothelial cell; IC, immune cell; FB, fibroblast. **f**, UMAP plot showing different cell types in the bone. EPC, endothelial progenitor cells; HSC, hematopoietic stem cell; DC, Dendritic cells; Mono, monocytes. **g**, UMAP plot showing different cell types in the human brain. L2/3IT, layer2/3 intratelencephalic projecting; L4IT, layer4 intratelencephalic projecting; L5IT, layer5 intratelencephalic projecting; L6IT, layer6 intratelencephalic projecting; L6ITCar3, layer6 intratelencephalic projecting Car3; L5ET, layer5 Extratelencephalic projecting; L5/6NP, layer5/6 Near-projecting; L6b, layer6b; L6CT, layer6 corticothalamic projecting; SST, SST+ neuron, LAMP5, LAMP5+ neuron; SST CHODL, SST+ CHODL+ neuron; PVALB, PVALB+ neuron; LAMP5 LHX6, LAMP5+ LHX6+ neuron; LAMP5, LAMP5+ neuron; SNCG, SNCG+ neuron; VIP, VIP+ neuron; PAX6, PAX6+ neuron; Astro, astrocytes; Oligo, oligodendrocytes; OPC, oligodendrocyte precursor cells; Micro, microglia; Endo, endothelialcells; VLMC, vascular leptomeningeal cells; PC, pericytes; SMC, smooth muscle cells; Immune, immune cells; RB, red blood lineage cells. **h,** UMAP plot showing different cell types in the skeletal MuSC. MF-I, type I myofiber; MF-II, type II myofiber; MF-Isn (fg), type I myofiber fragment from snRNA-seq; MF-IIsn (fg), type II myofiber fragment from snRNA-seq; MF-Isc (fg), type I myofiber fragment from scRNA-seq; MF-IIsc (fg), type II myofiber fragment from scRNA-seq; Specialised MF, specialised myonuclei and myocyte populations; Hyb, hybrid myofiber; MuSC, muscle stem cell; FB, fibroblast; EnFB, endoneurial fibroblast; PnFB, perineurial fibroblast; mSchwann, myelinating Schwann cell; nmSchwann, Non-myelinating Schwann cell; B-plasma, Plasma cell; cDC1, Conventional dendritic cell 1; cDC2, conventional dendritic cell 2; pDC, plasmacytoid dendritic cells; ArtEC, arterial endothelial cell; VenEC, venous endothelial cell; CapEC, capillary endothelial cell; LymphEC, lymphatic endothelial cell; SMC, smooth muscle cell; Pericyte, pericyte; RBC, red blood cell. **i,** UMAP plot showing different cell types in the myometrium. SMC, smooth muscle cell; PNS, peripheral nervous system cell; LEC, lymphatic endothelial cells.


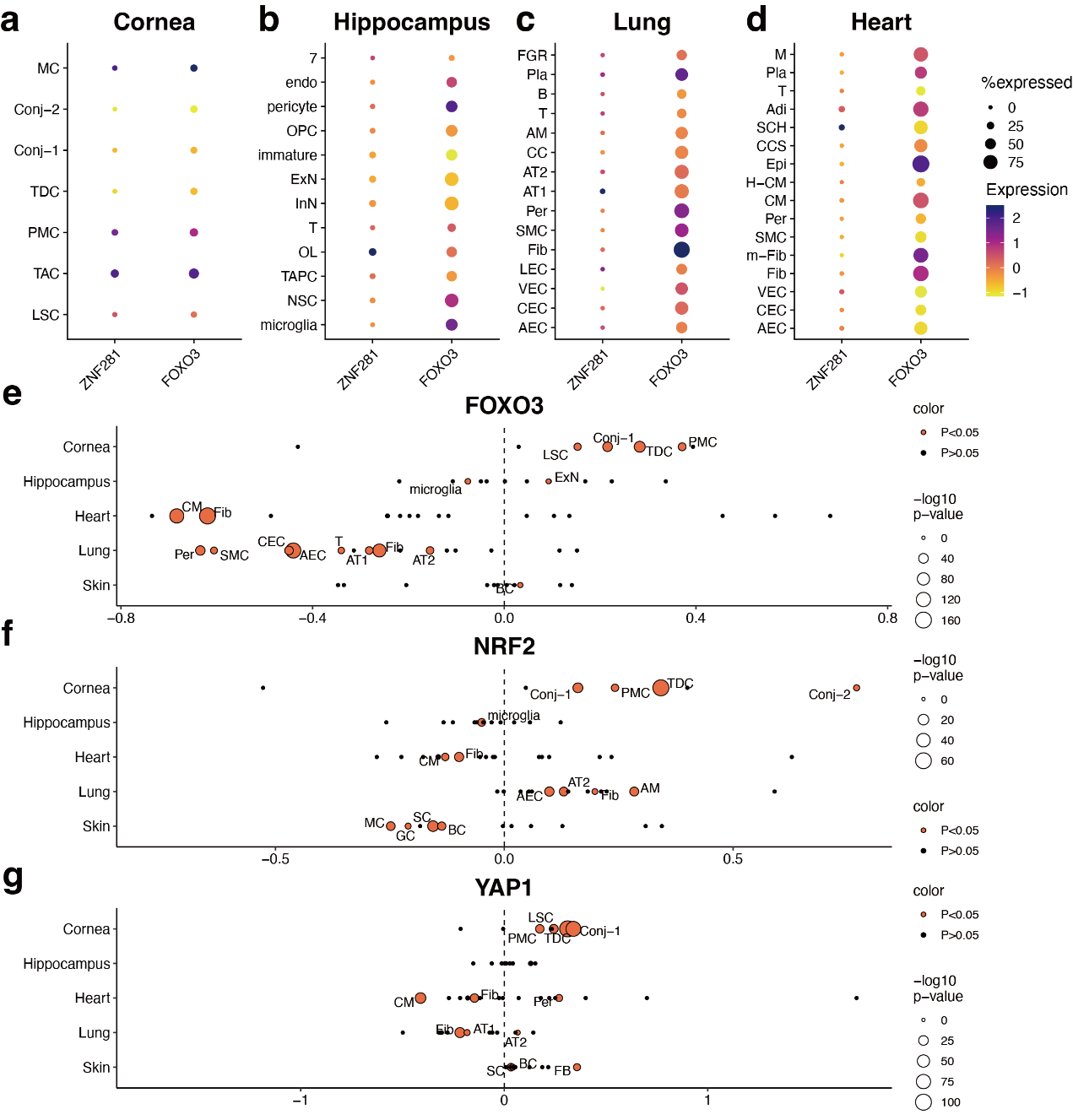


**Supplementary Fig. 3. The baseline expression and transcriptional change across aging monkey organs.** **a-d,** Dot plots showing the baseline expression level of ZNF281 and FOXO3 in young cornea (**a**), hippocampus (**b**), lung (**c**), and heart (**d**). **e-g,** Dot plots showing the variation in *FOXO3* (**e**)*, NRF2* (**f**) *and YAP1* (**g**) expression during aging across different organs. CM, cardiomyocyte; Per, pericyte; Fib, fibroblast; SMC, smooth muscle cell; CEC, capillary endothelial cell; AEC, arterial endothelial cell; AT1, alveolar type I cell; AT2, alveolar type II cell; ExN, excitatory neurons; BC, basal cell; LSC, limbal stem cell; Conj-1, conjunctival epithelial cells-1; Conj-2, conjunctival epithelial cells-2; TDC, terminally differentiated cell; PMC, post-mitotic cell; MC, melanocyte; GC, granular cell; SC, spinous cell; AM, alveolar macrophage.


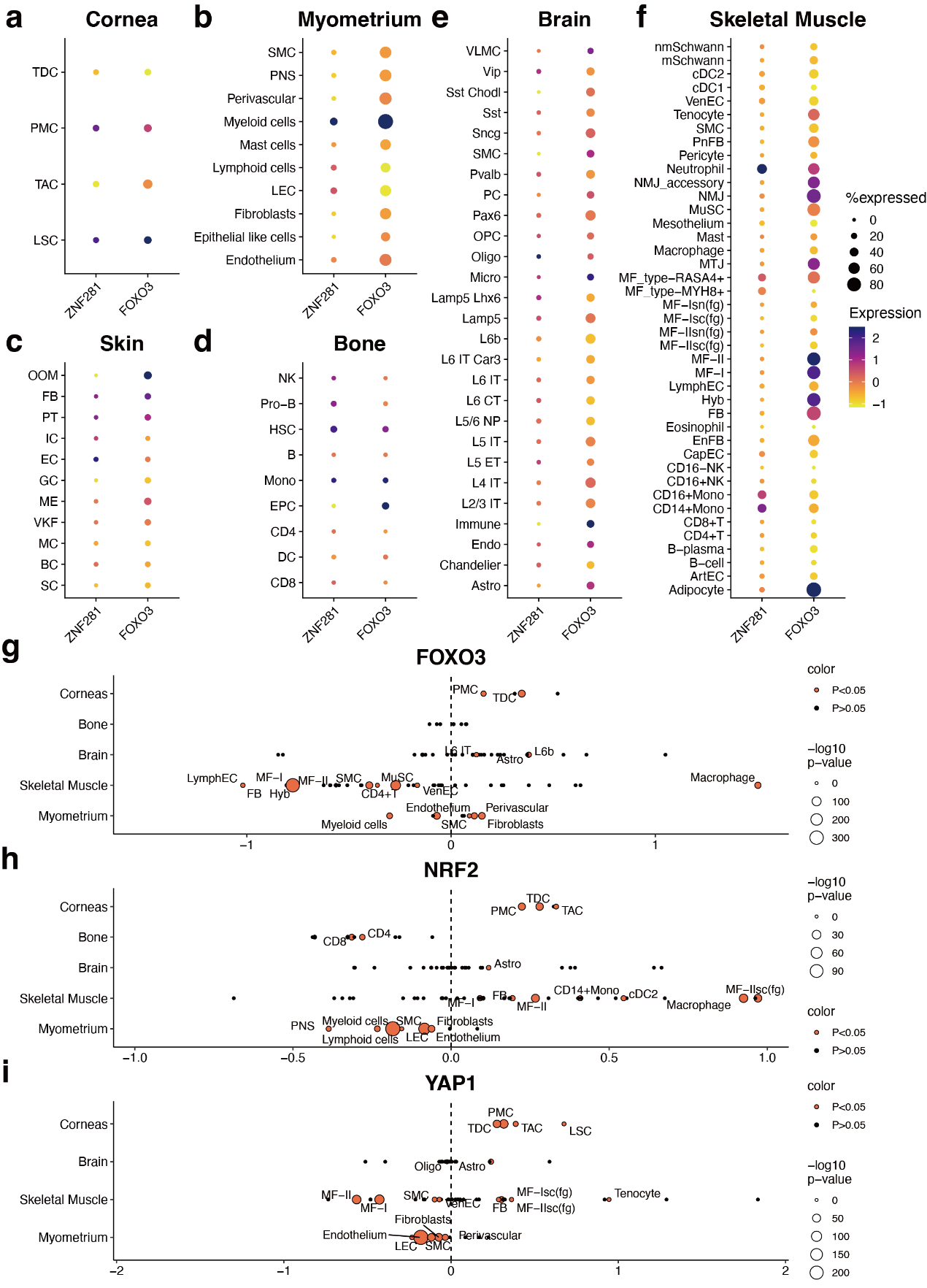
**Supplementary Fig. 4. The baseline expression and transcriptional change across aging human organs.** **a-d,** Dot plots showing the baseline expression level of ZNF281 and FOXO3 in young cornea (**a**), myometrium (**b**), skin(**c**), and bone (**d**), brain(**e**), and skeletal muscle (**f**). **g-i,** Dot plots showing the variation in *FOXO3* (**g**)*, NRF2* (**h**) *and YAP1* (**i**) expression during aging across different organs. LymphEC, lymphatic endothelial cell; FB, fibroblast; MF-I, type I myofiber; Hyb, hybrid myofiber; MF-II, type II myofiber; SMC, smooth muscle cell; MuSC, muscle stem cell; VenEC, venous endothelial cell; L6IT, layer6 intratelencephalic projecting; Astro, astrocytes; Oligo, oligodendrocytes; L6b, layer6b; PMC, post-mitotic cell; TDC, terminally differentiated cell; LEC, lymphatic endothelial cell; TAC, transit amplifying cell; cDC2, conventional dendritic cell 2; MF-Isc (fg), type I myofiber fragment from scRNA-seq; MF-IIsc (fg), type II myofiber fragment from scRNA-seq; LSC, limbal stem cell.


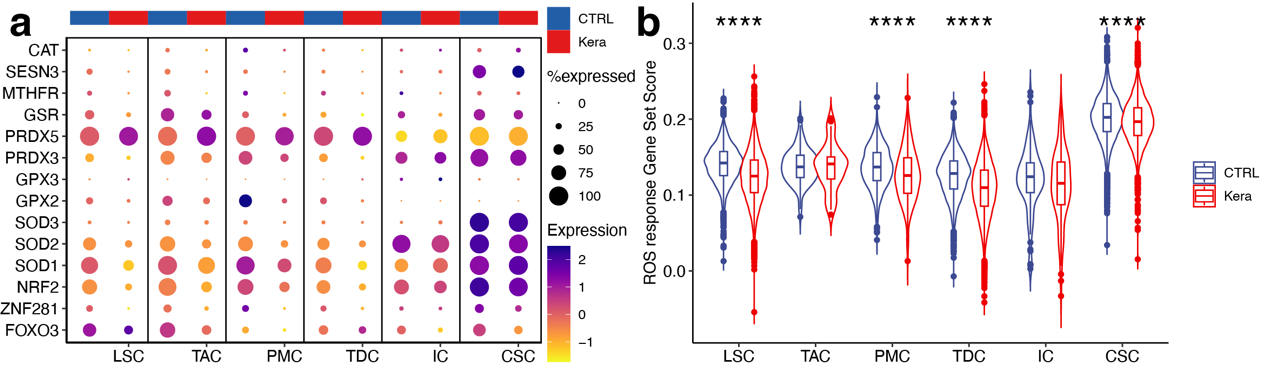


**Supplementary Fig. 5. Transcriptional alterations of ZNF281, FOXO3, NRF2, and their antioxidant target genes in keratoconus cornea.** **a,** Dot plot showing the differential expression of antioxidant genes in keratoconus cornea compared to normal cornea. IC, Immune cell; CSC, corneal stromal cell. **b,** Violin plot depicting the distribution of gene set scores for 'Response to ROS' in keratoconus and normal corneal cells.


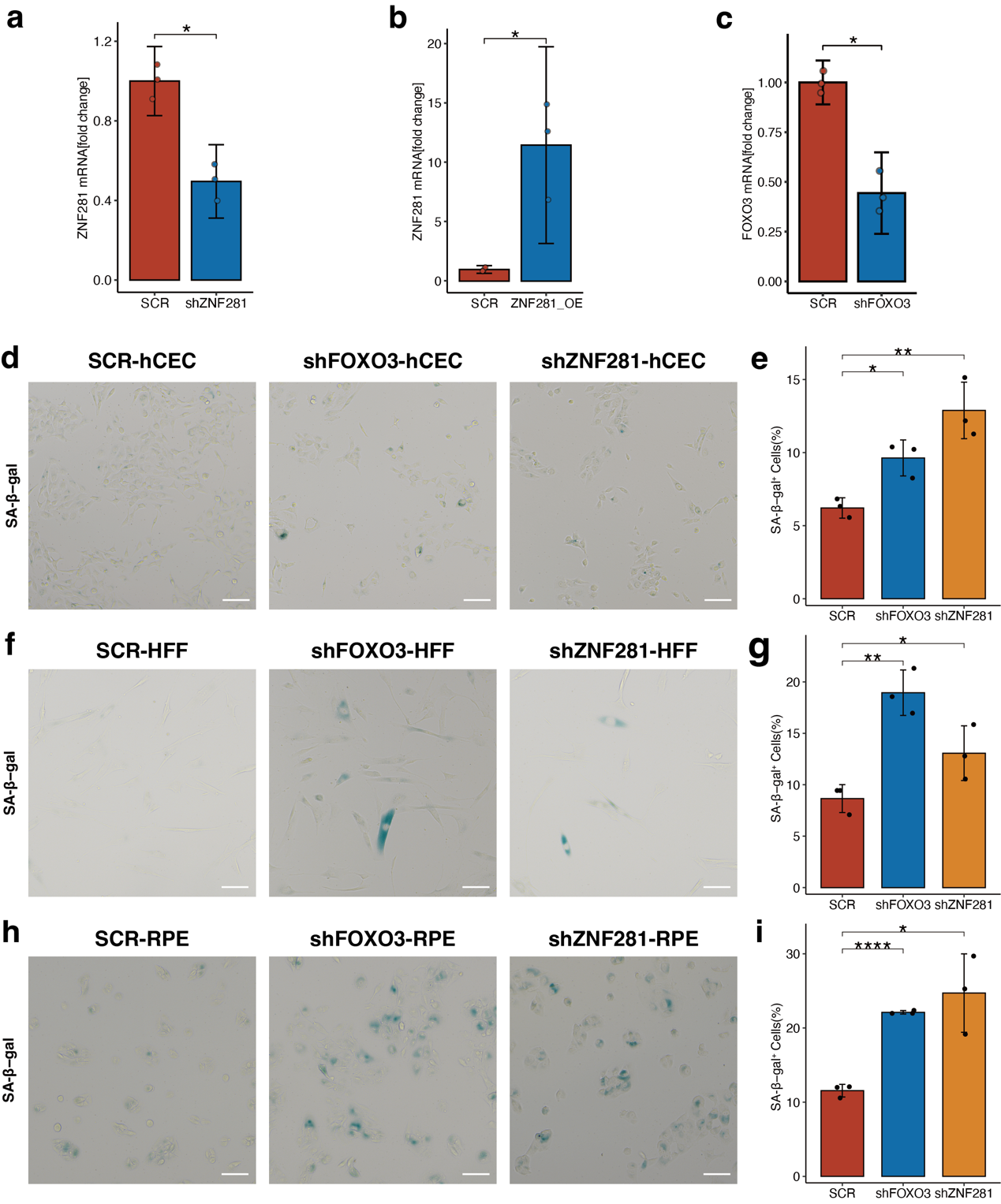


**Supplementary Fig. 6. Lentivirus-mediated knockdown of ZNF281 and FOXO3 significantly induces cellular aging in human cell lines. a-b,** Lentivirus-mediated overexpression **(a)** and knockdown **(b)** of ZNF281. Data are presented as the mean ± SEM. n = 3 for each group. *p <0.05. **c,** Lentivirus-mediated knockdown of FOXO3 **(c)**. Data are presented as the mean ± SEM. n = 3 for each group. *p <0.05. **d,** Representative micrographs showing SA-β-gal-positive cells among scramble, and FOXO3-knockdown and ZNF281-knockdown hCECs, Scale bars = 20 µm. **e,** Bar plots showing the increase in SA-β-gal-positive CEC number following FOXO3 and ZNF281 knockdown. Data are presented as the mean ± SEM. n = 3 for each group. *p <0.05; **p <0.01. **f,** Representative micrographs showing SA-β-gal-positive cells among scramble, and FOXO3-knockdown and ZNF281-knockdown HFF, Scale bars = 20 µm. **g,** Bar plots showing the increase in SA-β-gal-positive HFF number following FOXO3 and ZNF281 knockdown. Data are presented as the mean ± SEM. n = 3 for each group. *p <0.05; **p <0.01. **h,** Representative micrographs showing SA-β-gal-positive cells among scramble, and FOXO3-knockdown and ZNF281-knockdown RPE, Scale bars = 20 µm. **i,** Bar plots showing the increase in SA-β-gal-positive RPE number following FOXO3 and ZNF281 knockdown. Data are presented as the mean ± SEM. n = 3 for each group. *p <0.05; ****p <0.0001.


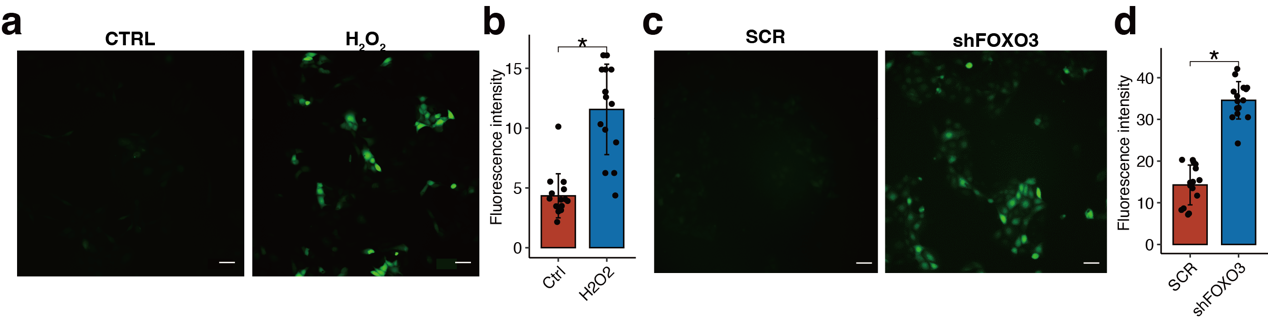


**Supplementary Fig. 7. H_2_O_2_-treated and FOXO3 knockdown accelerated the ROS levels in hCECs. a,** Representative micrographs showing ROS levels as measured by DCFH-DA fluorescence in control and H_2_O_2_-treated hCECs, Scale bars = 20 µm. **b,** Bar plot of mean fluorescence intensity in cultures treated as shown in (**a**). Data are presented as the mean ± SEM. n = 15 for each group. *p <0.05. **c**, Representative micrographs showing ROS levels as measured by DCFH-DA fluorescence in hCECs transfected with scramble control or FOXO3-targeted shRNA, Scale bars = 20 µm. **d**, Bar plot of mean fluorescence intensity in cultures treated as shown in (**c**). Data are presented as the mean ± SEM. n = 15 for each group. *p <0.05.


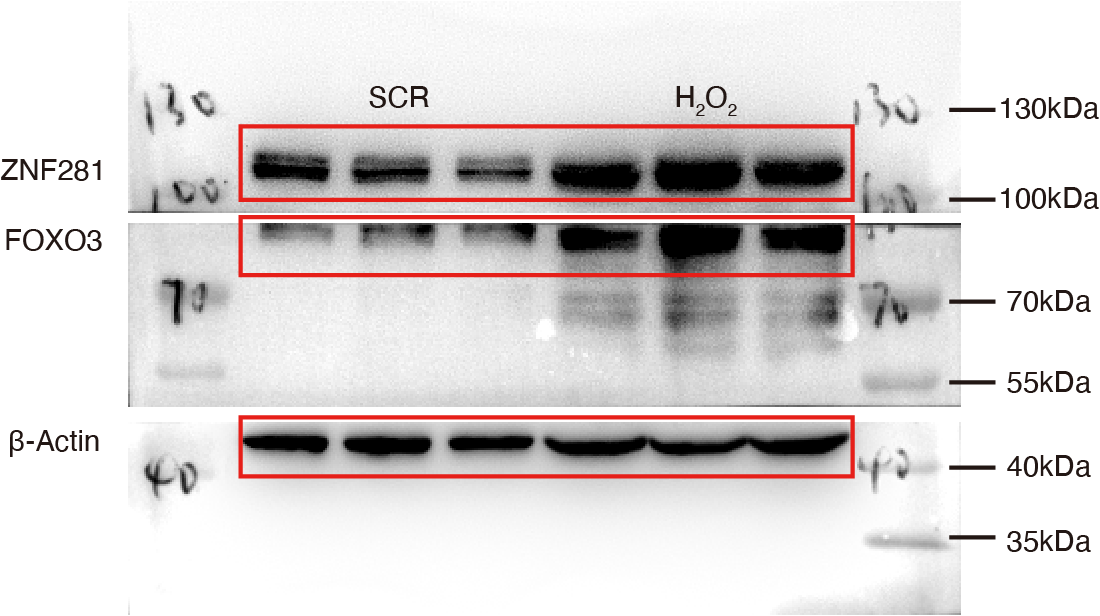


**Supplementary Fig. 8. Full scan (uncropped) western blots showing that ZNF281 and FOXO3 sense ROS levels.** Uncropped full scans of western blots from the **Fig. 3b** cropped western blots shown within the main text. Molecular weight markers are indicated.


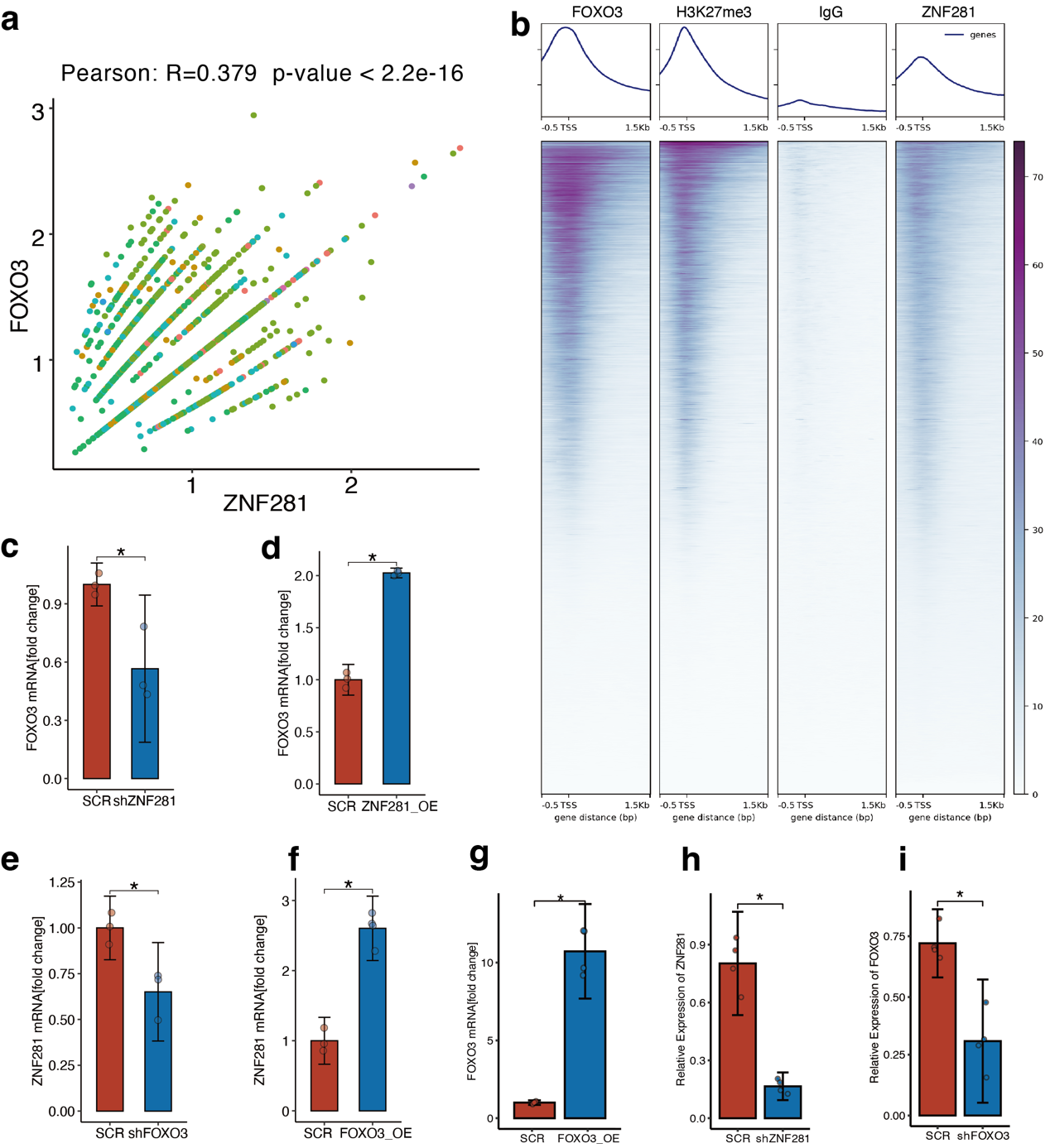


**Supplementary Fig. 9.** **Reciprocal** **regulation of ZNF281 and FOXO3. a,** Scatter plot showing the correlation between ZNF281 and FOXO3 expression at single cell level. **b,** Heatmap showing the intensities of FOXO3, ZNF281, IgG, and H3K27me3 from CUT&Tag experiments. **c-d,** Bar plots of qPCR results showing ZNF281 regulates the *FOXO3* expression. *ZNF281* knockdown decreased *FOXO3* expression (**c**), while *ZNF281* overexpression enhanced *FOXO3* expression (**d**). Data are presented as the mean ± SEM. n = 3 for each group. *p <0.05. **e-g,** *FOXO3* knockdown decreased *ZNF281* expression (**e**), while *FOXO3* overexpression enhanced *ZNF281* expression (**f, g**). Data are presented as the mean ± SEM. n = 3 for each group. *p <0.05. **h-i,** Bar plots showing significant decreases in ZNF281 and FOXO3 protein levels following shRNA transfection. Data are presented as the mean ± SEM. n = 4 for each group. *p <0.05.


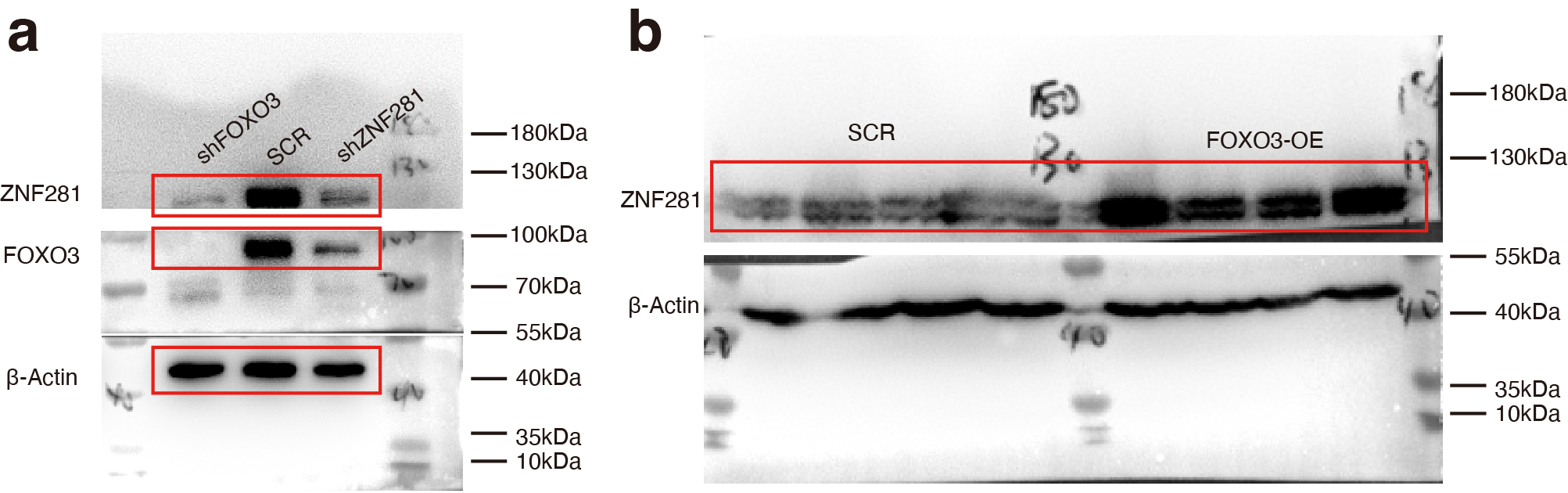


**Supplementary Fig. 10. Full scan (uncropped) western blots showing that ZNF281 and FOXO3 form a positive feedback loop.** Uncropped full scans of western blots from the **Fig. 4b** (**a**) and **Fig. 4f** (**b**) cropped western blots shown within the main text. Molecular weight markers are indicated.
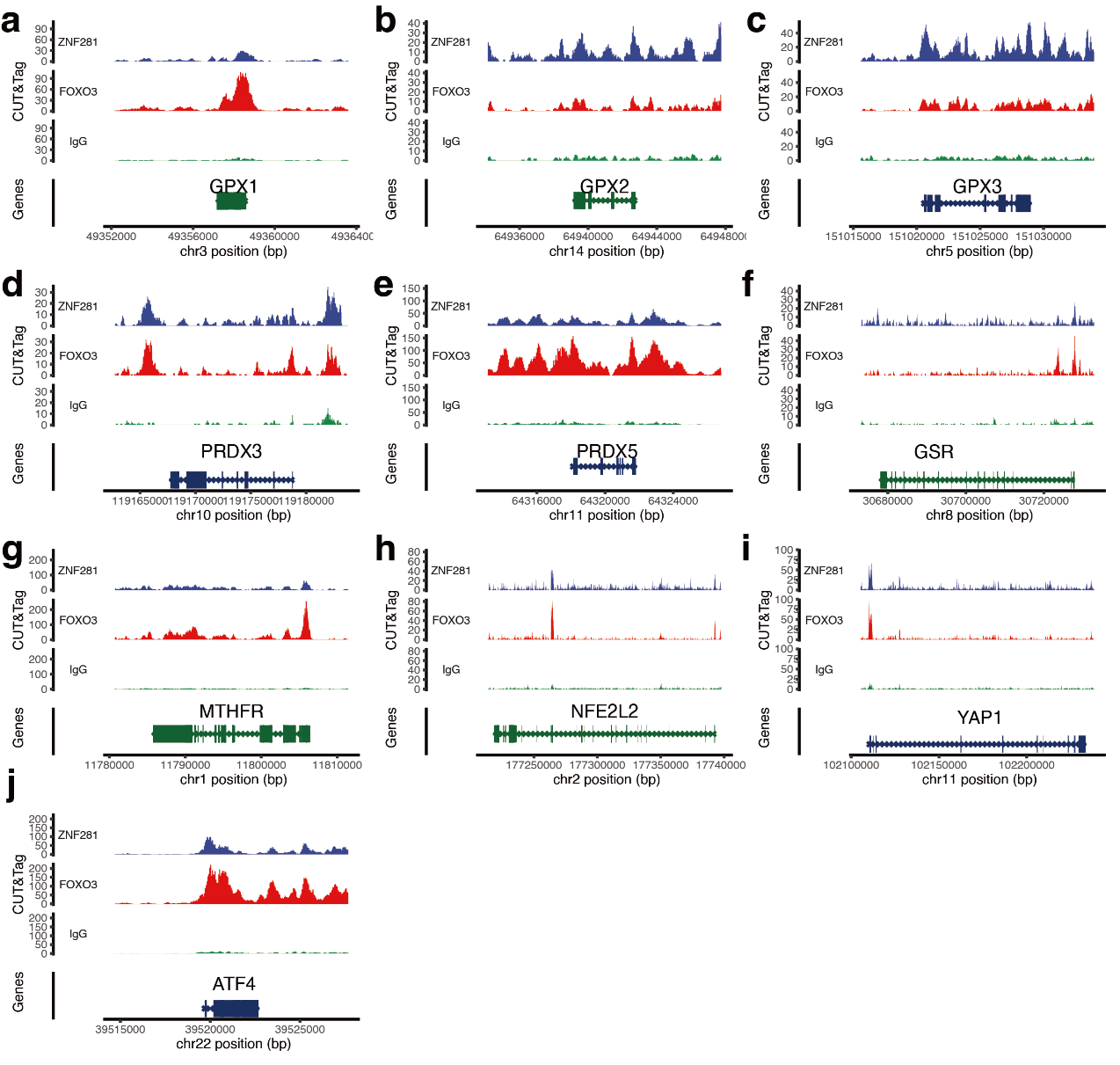


**Supplementary Fig. 11.** **ZNF281 and FOXO3 decrease ROS level by regulating the expression of antioxidant genes. a-j,** Track plots showing the binding of ZNF281 and FOXO3 to the promoter regions of antioxidant genes.


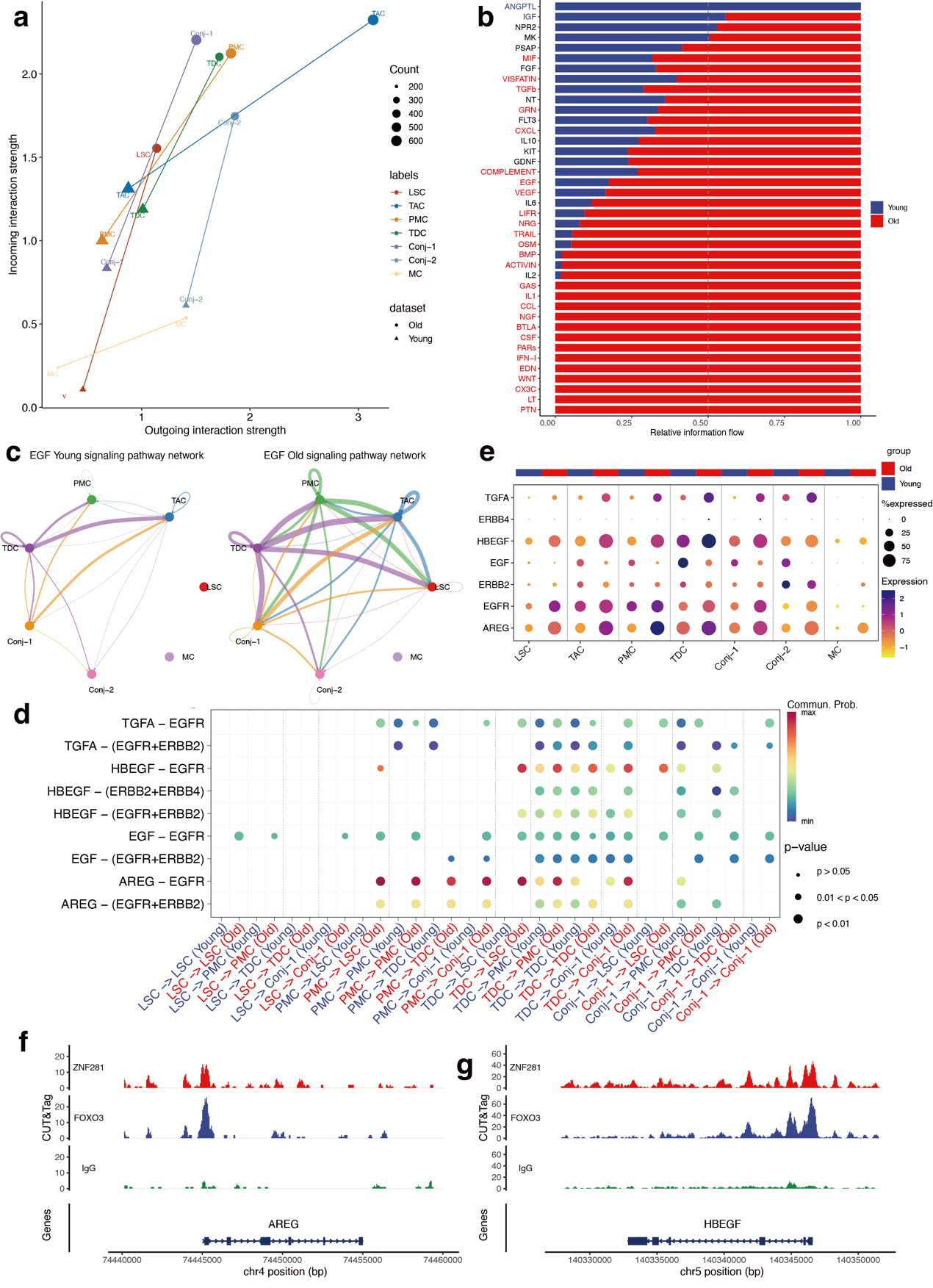


**Supplementary Fig. 12. Aging-associated changes in cell communication during the cornea aging process. a,** Outgoing and incoming interaction strength was enhanced in aging corneal epithelial cells. **b,** All significant signaling pathways were ranked based on their differences of overall information flow between old and young groups. The top signaling pathways, colored in red, are more enriched in the young group; the middle ones, colored in black, are equally enriched in both young and old groups; and the bottom ones, colored in red, are more enriched in the old group. **c,** The inferred EGF signaling networks in the corneal epithelium of young (left) and old monkeys (right). **d,** Comparison of significant ligand-receptor pairs of EGF signaling pathway between young and aging corneas. **e,** Expression distribution of genes involved in EGF signaling pathway. **f-g,** Track plots showing the binding of ZNF281 and FOXO3 to the promoter regions of AREG **(f)** and HBEGF **(g)**.


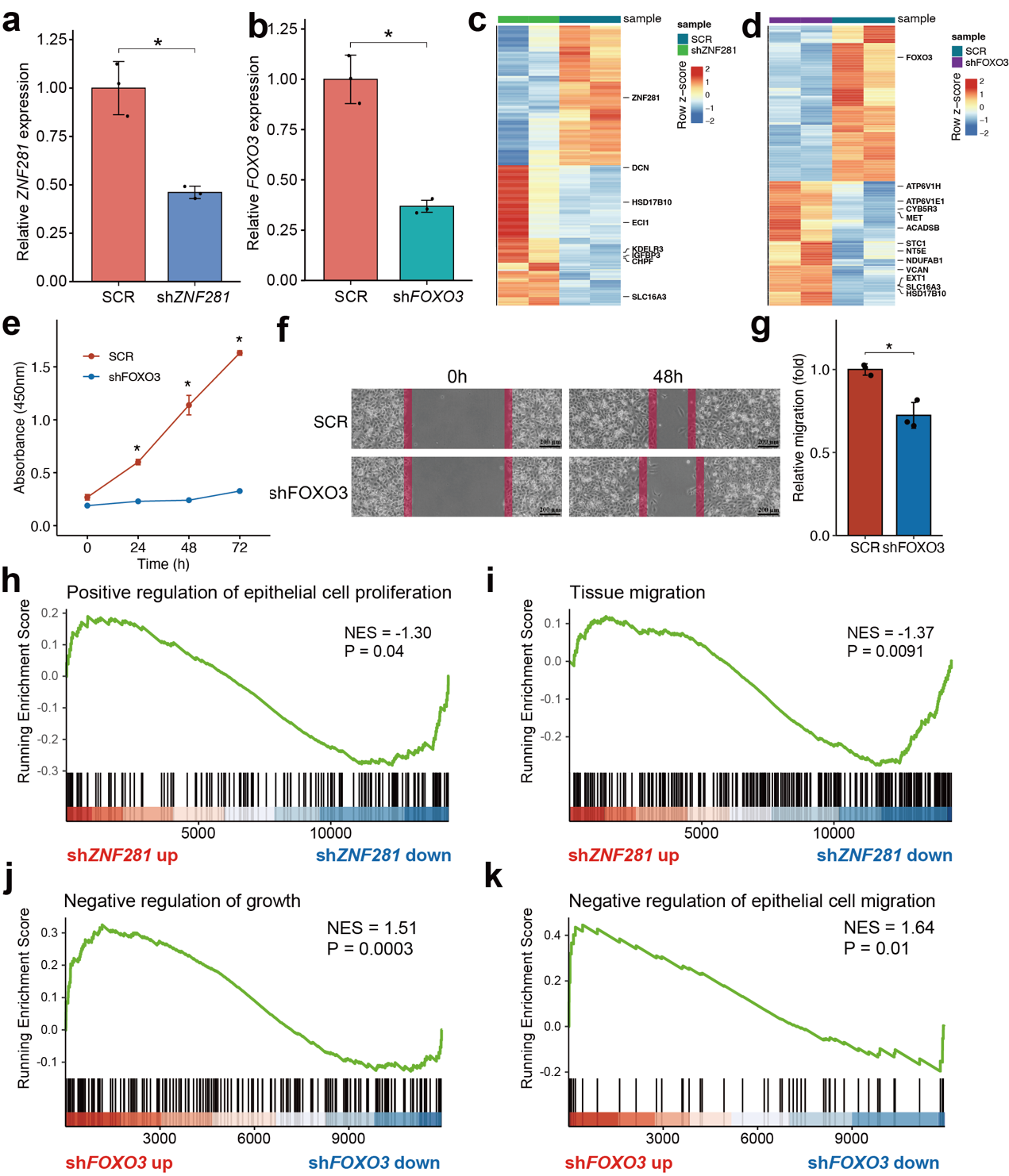


**Supplementary Fig. 13.** **Knockdown of ZNF281 and FOXO3 siginificantly upregulated the expression of metabolism-associated genes. a-b,** Bar plots showing the relative expression of *ZNF281* by hCECs upon infection with lentiviral shRNA vector targeting *ZNF281* (**a**) and *FOXO3* (b) upon infection with lentiviral shRNA targeting *ZNF281*. Data are presented as the mean ± SEM. n = 3 for each group. *p <0.05. **c-d,** Heatmaps of DEGs following *ZNF281* knockdown (**c**) and *FOXO3* knockdown (**d**).


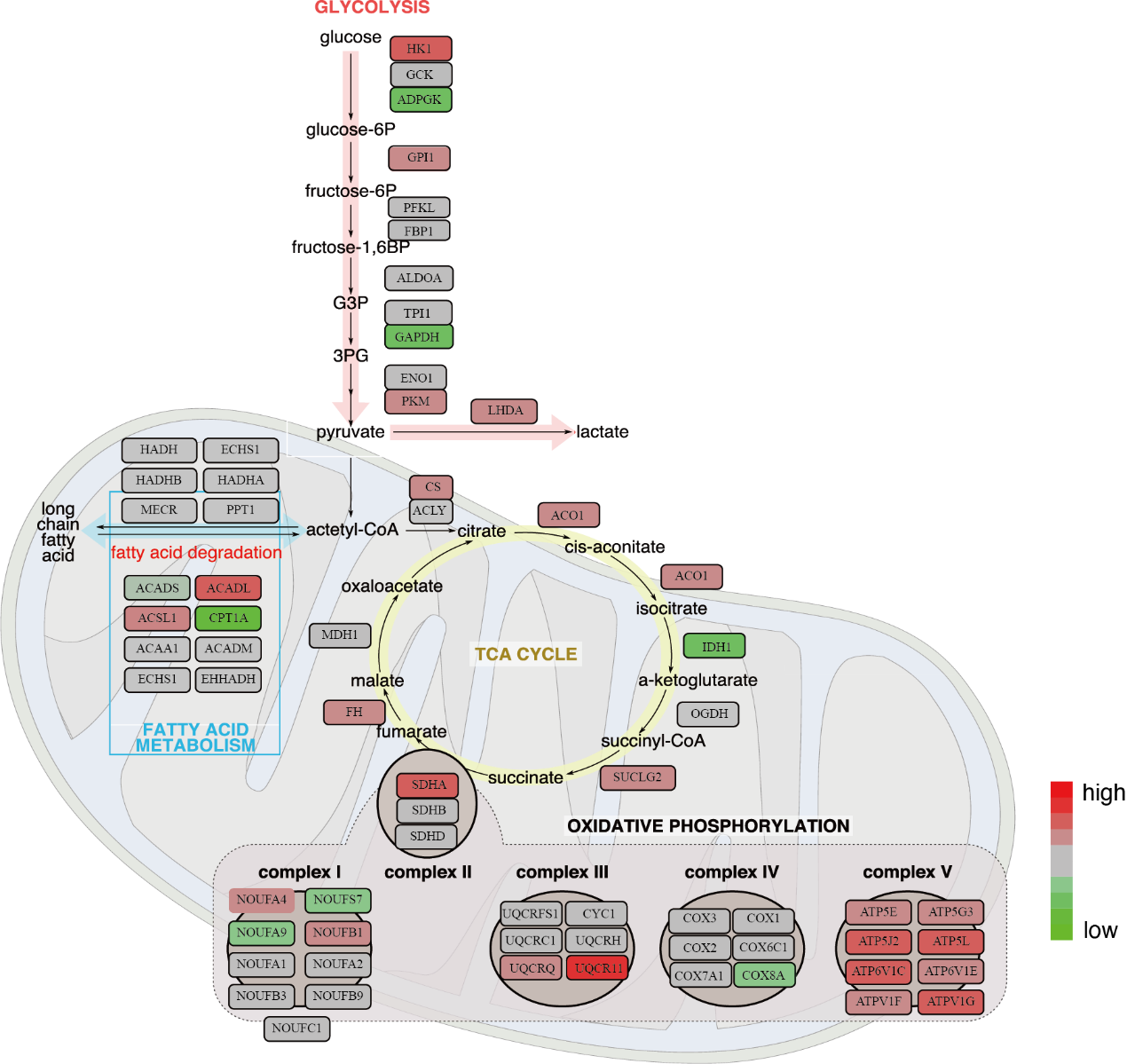


**Supplementary Fig. 14.** Heatmap showing the changes in expression of metabolism-associated genes by *FOXO3* knockdown.
